# Unveiling Arthropod Responses to Climate Change: A Functional Trait Analysis in Intensive Pastures

**DOI:** 10.3390/insects15090677

**Published:** 2024-09-07

**Authors:** Sophie Wallon, François Rigal, Catarina D. Melo, Rui B. Elias, Paulo A. V. Borges

**Affiliations:** 1 CE3C—Centre for Ecology, Evolution and Environmental Changes, Azorean Biodiversity Group, CHANGE—Global Change and Sustainability Institute, School of Agricultural and Environmental Sciences, University of the Azores, Rua Capitão João d’Ávila, Pico da Urze, 9700-042 Angra do Heroísmo, Portugalrui.mp.elias@uac.pt (R.B.E.); paulo.av.borges@uac.pt (P.A.V.B.); 2 Institut des Sciences Analytiques et de Physico Chimie Pour L’environnement et les Materiaux UMR 5254, Comité National de la Recherche Scientifque—University de Pau et des Pays de l’Adour—E2S UPPA, 64053 Pau, France; 3 CFE—Centre for Functional Ecology, Universidade de Coimbra, Calçada Martim de Freitas, 3000-456 Coimbra, Portugal; 4 IUCN SSC Atlantic Islands Invertebrate Specialist Group, 9700-042 Angra do Heroísmo, Portugal

**Keywords:** functional traits, arthropods, grassland, Azores, increased temperature, open top chambers, altitudinal gradient

## Abstract

**Simple Summary:**

Climate change is profoundly affecting ecosystem dynamics, with grassland arthropods serving as critical indicators. This in situ study examines the responses of arthropod communities to rising temperatures in intensively managed pastures on the volcanic island of Terceira (Azores, Portugal), employing a functional trait approach. Along an elevation gradient, Open Top Chambers (OTCs) were used to simulate increased temperatures, allowing the analysis of soil-dwelling arthropods over winter and summer seasons. Our results underscore the nuanced responses of arthropods to temperature variations across elevations and treatments within intensive pastures, revealing significant changes in functional community composition. This study emphasizes the importance of considering functional traits when assessing patterns of diversity in complex ecological communities involving multiple trophic levels.

**Abstract:**

This study investigates the impact of elevated temperatures on arthropod communities in intensively managed pastures on the volcanic island of Terceira, Azores (Portugal), using a functional trait approach. Open Top Chambers (OTCs) were employed to simulate increased temperatures, and the functional traits of ground dwelling arthropods were analyzed along a small elevation gradient (180–400 m) during winter and summer. Key findings include lower abundances of herbivores, coprophagous organisms, detritivores, and fungivores at high elevations in summer, with predators showing a peak at middle elevations. Larger-bodied arthropods were more prevalent at higher elevations during winter, while beetles exhibited distinct ecological traits, with larger species peaking at middle elevations. The OTCs significantly affected the arthropod communities, increasing the abundance of herbivores, predators, coprophagous organisms, and fungivores during winter by alleviating environmental stressors. Notably, iridescent beetles decreased with elevation and were more common inside OTCs at lower elevations, suggesting a thermoregulatory advantage. The study underscores the importance of considering functional traits in assessing the impacts of climate change on arthropod communities and highlights the complex, species-specific nature of their responses to environmental changes.

## 1. Introduction

Climate change has emerged as a defining challenge on a global scale, reshaping ecosystems and challenging the ability of different species to adapt [1,2]. In the context of these changes, arthropods as a taxonomic group are emerging as particularly sensitive indicators of environmental change [3,4]. These tiny but ecologically important organisms play a key role in maintaining ecosystem balance and are highly sensitive to changes in temperature, precipitation, and other environmental variables [5,6]. Thus, arthropods play a crucial role in ecosystem dynamics, reflecting the complex interplay between climate change and biological responses [7].

As temperature patterns shift and precipitation becomes more erratic [8,9], arthropods in different habitats are subject to disruptions in their abundance, distribution, and life cycles [10]. Understanding the impact of climate change on these arthropods is crucial not only for biodiversity conservation, but also for disentangling wider ecological consequences.

The global dimension of this issue enhances its importance. As indicators of environmental health, arthropods provide valuable insights into the wider effects of climate change [11]. Studying their responses to these changes can reveal patterns that transcend geographical boundaries, contributing to a more comprehensive understanding of the ecological consequences of climate change on a global scale [12,13].

Within this global panorama, however, our study focuses on a more specific investigation—the realm of pastureland arthropods in the volcanic archipelago of the Azores. Intensive managed pastures represent more than 56% of the land occupation in this region [14]. These islands host a unique diversity of arthropods, with most endemic species being currently restricted to mid-to-high-elevation native forests [12,13]. By narrowing our focus to a particular ecological niche (e.g., pastureland), we aim to unravel the nuances of climate change impacts on a specific group of arthropods associated with intensively managed pasture, providing insights that can be extrapolated to similar ecosystems worldwide.

The pastures in the Azores serve as habitats for various arthropod species from different origins (indigenous and non-indigenous) [15,16,17,18,19]. The interdependence between these arthropods and the grassland ecosystem is important for the good health of the pastures [20,21,22]. As climate change affects the Azores with temperature increases and changes in precipitation patterns (stronger events during the winter and longer periods of drought through the summer) [23,24], it poses unprecedented challenges for agriculture. In accordance with the findings presented in [14], the primary impacts linked to climatic factors in the Azores revolve around induced stress and diminished agricultural production during drought conditions. Additionally, there is notable evidence of reduced pasture and fodder quality, accompanied by the expansion of certain insect pests.

In the context of the Azores, temperatures are expected to increase by a value between 0.78 °C and 0.90 °C by 2039. Looking further ahead, to the end of the century, temperature projections indicate an increase of between 1.5 °C and 2.8 °C, corresponding to the two different scenarios, RCP4.5 and RCP8.5 [14]. 

The Azores, with their isolated and distinct ecological context [25,26], face both challenges and opportunities because of climate change. Shifts in temperature and precipitation pose a threat to the stability of arthropod communities [27], while, at the same time, opening avenues for understanding their resilience and adaptive strategies. 

To unravel the specific impacts of climate change on arthropods in the Azores, our study used Open Top Chambers (OTCs) as a controlled experimental tool along an altitudinal gradient. OTCs provide a unique opportunity to simulate temperature increases, allowing us to mimic expected climate scenarios [28]. First, this controlled environment allows for a focused study of how the arthropod communities within the pastures respond to elevated temperatures [29,30], a key facet of climate change. The use of OTCs aims to bridge the gap between observational studies and experimental manipulations [31,32], which should provide a more nuanced understanding of the direct effects of temperature change on arthropod functional traits in the Azores. Secondly, the inclusion of a small elevation gradient (180–400 m) in experiments on the effects of climate change on arthropod communities is crucial, as it provides natural variations in temperature and environmental conditions, allowing researchers to study species and community responses to these changes in a controlled setting [33,34]. The combination of OTCs and an elevation gradient is essential for understanding how climate change might alter biodiversity patterns and ecological interactions across treatments and elevations. 

Traditionally, species community monitoring has relied on the application of diversity indices based on taxonomic classifications [35]. These indices, such as the Shannon Diversity Index and species richness, provide valuable metrics for assessing the diversity and evenness of species within an ecosystem [36]. However, while taxonomic approaches provide a snapshot of community composition, they may miss subtle shifts in functional dynamics and ecological interactions [37]. Our previous study acknowledged the utility of these traditional metrics [30], but recognized the need for a more comprehensive approach to capture the nuanced responses of arthropod communities to climate change. By incorporating functional traits into our analysis, we aim to overcome the limitations of taxonomy-based assessments and provide a more holistic understanding of how arthropods are responding to changing environmental conditions in Azores pastures. This shift in methodology allows us to uncover functional nuances that may be obscured by traditional taxonomy-based alpha diversity measures (e.g., species richness, the Shannon Index), ultimately enriching our understanding of the ecological impacts of increased temperature on arthropod communities.

Functional traits, which include morphological and behavioral characteristics, provide a more dynamic lens through which to examine species’ responses to changing climate or disturbances [38,39,40]. The importance of this different approach lies in its ability to unravel how arthropod communities function within the ecological framework of Azores pastures under climate change. By focusing on functional traits, we can decipher not only which species are present, but also how they interact with their environment, providing a more nuanced understanding of the ecological consequences of temperature increase. 

We aim to determine how increasing temperatures (simulating climate-induced changes) along a short elevation gradient manifest themselves in a functional trait approach to arthropod communities within intensively managed pastureland. To this end, we seek to identify key functional traits that serve as indicators of adaptation or vulnerability in the context of temperature increase.

1. We posit that different guilds of arthropods within pastureland communities will display varying responses to temperature changes. Specifically, we anticipate that predator-and-prey relationships, as well as the presence of herbivores, will be reshaped under increased temperatures due to grass biomass availability. Arthropod guilds may respond differently to temperature shifts along the elevational gradient and between treatments due to their distinct ecological roles and specific traits. Predators, for instance, may experience changes in prey availability or altered hunting efficiencies (decreases in predators in the warmest sites: OTCs and lower altitudes), while herbivores may face shifts in plant suitability along the gradient, the treatment, and seasons [41].
2. We hypothesize that as temperatures increase in pastureland ecosystems, there will be a specific pattern in the distribution of arthropod species’ body sizes, with smaller species in the warmest sites (OTCs and lower altitudes) [42].
3. Beetles’ color and iridescence may influence their thermoregulation and capacity to support increased temperatures. Darker beetles should prevail in cooler sites, while iridescent beetles may be more abundant in warmer sites [43,44].

## 2. Materials and Methods

### 2.1. Study Area

**Figure 1:**
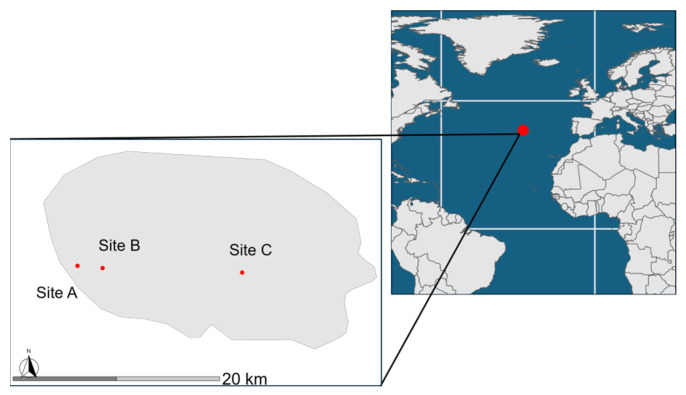
Localization of the island of Terceira and experimental sites.

The research was carried out on three experimental intensively managed pastures on the island of Terceira, part of the Azores archipelago in Portugal. The island of Terceira covers an area of 402 square kilometers and reaches a maximum altitude of 1023 m. Its geographical coordinates are between 38°37′ N and 38°48′ N latitude and 27°02′ W and 27°23′ W longitude. The sites, designated A, B and C, which are of low, middle, and high elevation, are located at different heights above sea level: site A at 186 m, site B at 301 m, and site C at 386 m. Their specific latitudes and longitudes are 38.703596° N, −27.353805° W for Site A, 38.701639° N, −27.325783° W for Site B, and 38.697770° N, −27.170075° W for Site C (Figure 1.). Each of these sites is classified as intensive pasture land. The primary vegetation consists of Italian ryegrass, *Lolium multiflorum* Lam. (Poaceae), in Sites A and B, and common velvet grass, *Holcus lanatus* L. (Poaceae), in Site C.

### 2.2. Experimental Design

A field experiment was established using Open Top Chambers (OTCs). OTCs are common tools used in climate change research to manipulate abiotic environmental conditions directly in the field to mimic an increase in temperature [28] (Aronson and McNulty 2009). The structure of OTCs includes panels that act as wind barriers, effectively reducing heat loss by convection. In addition, their open-top design allows rain to enter aronsand air to circulate freely, resulting in the creation of small air vortices or eddies [32] (Hollister et al. 2022).

Within each field, twenty plots measuring 1 × 1 m were arranged in a grid layout, while maintaining a distance of 1.5 m between them. Half of these plots, selected at random, served as a control group, while the remaining ten were enclosed by OTCs. To monitor temperature variations, data loggers (Easy Log: EL-USB-2) were installed both inside the OTCs and within the control plots. On average, temperatures within the OTC-enclosed plots were found to be 1.2 °C higher than those in the control plots. The OTCs were designed to encompass the 1 × 1 m plots, as well as an additional 25 cm border around each plot. This buffer area allowed for easy access to the plots for maintenance and monitoring without causing damage and facilitated the placement of pitfall traps at each outer corner of the plot, still within the boundaries of the OTCs. Additionally, the OTCs were elevated approximately 5 cm off the ground to permit the unimpeded movement of crawling arthropods throughout the entire sampling area.

Sampling took place in two different seasons, winter and summer 2020, and was carried out before any mowing of the grass. Cattle access to the sampling plots was not possible, since fences were implemented. Most importantly, the experimental design, which included both control plots and plots enclosed by OTCs, was maintained throughout the year. Consequently, there was no relocation of the OTCs and control plots between the two sampling periods.

### 2.3. Arthropod Sampling and Identification

In this research, we concentrated on the impact of intensive pasture management on arthropod communities, with a specific focus on crawling arthropods. This focus was necessitated by the fact that OTCs act as barriers to flying insects, potentially skewing results towards these ground-dwelling species. To facilitate sampling, pitfall traps were employed, specifically targeting epigean arthropods. Across each plot, four pitfall traps were positioned at every corner, resulting in a total of 80 traps per field. These traps, consisting of a 330 mL plastic cup with dimensions of approximately 12 cm in depth and 8 cm in diameter at the opening, were filled with a 20% solution of ethylene glycol from car cooling liquid and a few drops of soap to reduce water surface tension. To protect the traps from rain and prevent overflow, they were covered with a plastic dish secured by small iron sticks, allowing unobstructed access to the traps. The collected specimens were preserved in 96% ethanol.

For analysis purposes, the data from the four traps at each plot were combined into a single sample, yielding 10 replicates for each treatment type across the fields: ten for control and ten for OTCs. Arthropod collection was executed in the winter and summer of 2020, with the traps set for 14 days, except for one instance, in Field B, during summer, where the traps were set for 13 days—here, data for the 14th day were extrapolated based on the other days’ findings. However, species richness estimates were not extrapolated.

All collected arthropods were sorted and identified to the species level when feasible, covering target groups such as Arachnida (Araneae, Opiliones, Pseudoscorpiones), Diplopoda, Chilopoda, and most Insecta groups, including Formicidae and Lepidoptera, but excluding Diptera and Hymenoptera. Unidentified specimens were given a morphospecies code. The initial sorting and identification tasks were carried out by the lead author (SW) and assisting students (acknowledged as parataxonomists), followed by verification by an expert taxonomist (PAVB). Species nomenclature and colonization status followed the latest checklist of Azorean arthropods [45].

It is noteworthy that, in Terceira’s intensive pastures, exotic arthropods typically dominate, with native and/or endemic species’ presence diminished due to significant land disturbance [38,46]. Consequently, no distinction was made regarding the biogeographical origin of species in our analyses. Despite previous Azorean arthropod studies often including juvenile spiders (e.g., [46]), the prevalence of Erigoninae linyphiid spiders in this study posed challenges to adopting such an approach. All specimens are stored in the Dalberto Teixeira Pombo (DTP) Collection at the University of the Azores (Terceira Island), with data accessible in [18] through a specified URL.

### 2.4. Functional Traits

We gathered data on body size and dispersal ability for each arthropod species studied. We also compiled a collection of functional traits related to resource use. Finally, for beetles, specific traits were gathered regarding their color and presence or absence of iridescence (Table 1).

Information on traits was gathered through a thorough review of the literature, encompassing original species descriptions, initial records of species in the Azores, short communications, and ecological research. Additionally, information was obtained from experts who identified the specimens, or specialists (P.A.V. Borges, Pedro Cardoso, Sofia Terzopoulou) in the relevant taxonomic group, in cases where specific species information was unavailable.

Functional traits for each species were determined based on the characteristics of the adult stage. For morphospecies that could not be identified to species level, we assumed functional traits based on the closest taxonomic classification available, such as genus or family. Details of these trait assignments for each species can be found in Tables S1 and S2.

### 2.5. Data Analysis

Data from the four traps in each plot were grouped into a single value for analysis. During the winter, in four sampling events, one of the four traps was damaged due to flooding or earth filling. In these cases, we estimated the theoretical abundance of four traps by extrapolating from the data collected by the remaining three traps. Similarly, in cases in which traps were only operated for 13 days instead of the planned 14, we adjusted the sampling period by extrapolating arthropod abundances to cover the full 14 days. In situations in which either trap was damaged or the sampling period was reduced by one day, we assumed that species richness remained unchanged; only arthropod abundances were extrapolated.

Each trap was assigned a habitat, Field A, B or C, at low, middle, and high elevation, respectively, a treatment (control or OTCs), and a location (plot located on the edges or in the center of the field). All analysis were performed with R software (4.2.3) [47].

The analyses were carried out for winter and summer separately, considering all arthropods collected and only the most diverse taxon, namely the beetles (Insecta, Coleoptera).

For all arthropods, the traits analyzed were the following: the trophic guild (herbivorous, predators, fungivores, detritivores, and coprophagous organisms), how arthropods ingest food, e.g., feeding behaviors (piercing/sucking, chewing/cutting, and external digestion and sucking), some ecological traits, such as the daily activity (diurnal, nocturnal or active during twilight), the dispersal abilities (high or low dispersal abilities), and the standardized body size. 

Body size standardization was applied for each order present in our samples for analyses, considering all arthropods as follows:

Standardized body size of Species *x* = (body size of Species × Order average body size)/Order Standard Deviation body size

For beetles, the traits analyzed were the following: the trophic level (herbivorous, predators, fungivores, and detritivores), the beetle’s coloration, the presence or absence of iridescence and some ecological traits, such as the daily activity (diurnal, nocturnal or active during twilight), high dispersal abilities, and the real body size. Feeding behaviors were not analyzed, as all beetles feed by chewing and cutting, and it was thus not a relevant functional trait to incorporate. Additionally, coprophagous trophic level and low dispersal abilities were not analyzed for beetles due to the lack of sufficient data to perform the analysis (only 3 species sharing those traits).

For each trait, we calculated the community weighted means (CWM) using the “functcomp” function from the FD package [48]. The CWMs express the mean attribute value between species occurring at a site, weighted by the relative abundance of each species [49], and were calculated as follows:

$${CWM}_{j}=\sum_{i=1}^{S}p_{i}x_{ij}$$

where CWM*j* is the community-weighted mean value of trait attribute *j*, *p_i_* is the relative abundance of species *i* (*i* = 1, 2, …, S), and x*_ij_* is the value of trait attribute *j* for species *i*. Prior to the CWM computation, abundance data were square-root-transformed to reduce the influence of highly dominant species. Nominal traits were dummy-transformed to as many binary variables as there were trait attributes. All binary traits were then treated as continuous variables using the “bin.num” parameter in the function “functcomp”.

The resulting CWM values for both seasons were combined with habitat, location, and treatment information to form a comprehensive data frame. The relationships were visualized using boxplots created with the ggplot2 package, highlighting differences by field, location, and treatment conditions.

A series of analyses of variance (ANOVA) was conducted to investigate the effects of elevation, location, and treatment on each trait, including the interaction terms on each CWM variable and for each season. When the overall ANOVA model was statistically significant, post hoc pairwise comparisons were performed using the “lsmeans” package to determine differences between levels of significative factors and/or interaction. 

Preliminary tests indicated only four traits for all arthropods and four traits for beetles responding with low significances for the effect of the plot location (edges or center) with ANOVA, and, according to post hoc pairwise comparison, of the few traits responding to the effect of plot location, most were not significant. Thus, we restricted our ANOVA to the fundamental effects of our design, i.e., field, treatment, and interaction between the two.

Because several tests were performed for each season, we decided to correct the P-values using the False Discovery Rate method (FDR, [50]) to guard against inflation of Type-I errors.

## 3. Results

In total, over the three fields and two seasons (winter and summer), we collected 41,351 specimens. For the current analysis involving functional traits, only adults specimens were considered, giving a total of 35,735 arthropods from four classes, 15 orders, 60 families, and 171 morphospecies. From these, 34 taxa were identified only at the order, family, or genus level, resulting in 137 taxa with associated scientific species names (n = 32,821), which will be referred to as ‘species’ from here on (see full data in [18]). 

**Figure 2:**
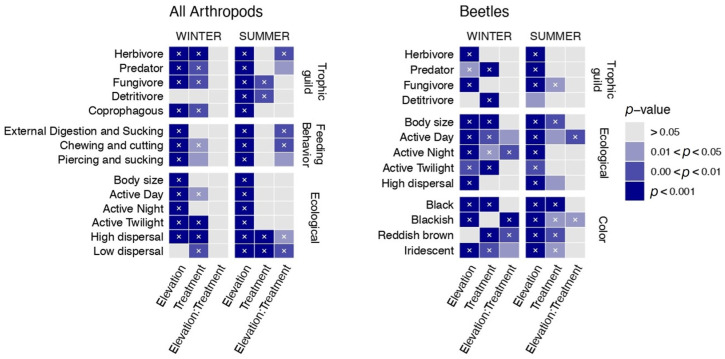
Heatmap for winter and summer season, highlighting the results of the analysis of variance (ANOVA), considering all arthropods and only beetles, which was conducted to investigate the effects of elevation, treatment, and their interaction (Elevation:Treatment) on each trait community weighted means (CWM) variables. On the (**left**) *y*-axis are the different traits analyzed. IngEDS: external digestion and sucking; IngCC: chewing and cutting; IngPS: piercing and sucking; Hdisp: high dispersal ability; Ldisp: low dispersal ability; ActDay: diurnal; ActNig: nocturnal; ActTw: active during twilight. On the (**right**) *y*-axis are the different categories of traits analyzed. The *x*-axis presents the different factors analyzed: elevation, treatment, and their interaction (Elevation:Treatment). The colors indicate the level of significance (dark blue, *p* < 0.001; medium blue, 0.00 < *p* < 0.01; light blue, 0.01 < *p* <0.05, and gray, *p* > 0.05). Cells marked with a “×” mean that the effect remained significant after the FDR correction for multiple testing. Results of the ANOVA are detailed in Table A1 and Table A3.

Our CWM analyses (Figure 2) show that the most important and strongest effect on all the functional traits studied is the factor field (e.g., elevation gradient) during both seasons for all arthropods and beetles. The second most important factor influencing functional traits is the treatment (control and OTC), i.e., elevated temperature. For all the arthropods, increased temperature had a greater effect during winter, especially on the ecological traits and guilds. Feeding behavior was not affected by the presence of OTCs. For the beetles, increased temperature had an effect on all the functional traits in both seasons. However, the effect was stronger in winter than in summer.

Finally, the interaction between field and treatment was the third main effect, and it showed that the effect of climate change varied according to the elevation. For all the arthropods, the interaction “field/treatment” showed an effect only in summer, mainly on feeding behavior and dispersal ability.

For the beetles, the interaction between field and treatment had a very small effect on diurnal activity, and for blackish beetles, this occurred in summer. In winter, color traits and diurnal and nocturnal activity were the most responsive traits. The results with the adjusted p-values were very similar, with only a few decreases with some significance. However, this did not change the main pattern of the results, confirming what was previously established.

With this context in mind, let us delve into the details. 

### 3.1. Winter and Summer Traits Analysis for All Arthropods

#### 3.1.1. Guilds

**Figure 3:**
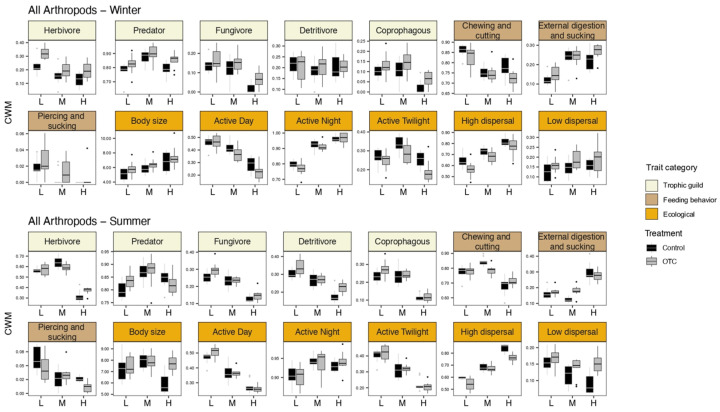
Boxplots highlighting the effect of the treatment (control vs. OTCs) on different traits along the elevation gradient considering all arthropods during the winter and the summer. On *y*-axis are the values of the community weighted means (CWM) and on *x*-axis is the elevation factor: low (L), middle (M), and high (H) elevation. Black box plots indicate the control plots, while gray box plots represent the OTCs. ANOVA and post hoc pairwise comparisons results are available in Table A1 and Table A2.

Considering all the arthropods during the summer, all the guilds (Figure 3) showed significantly lower abundances at high altitude than at middle and low altitude. There is an exception for predators, for which the highest abundance was found at mid-altitude for both seasons, showing a hump shape along the altitudinal gradient. During winter, the herbivores, coprophagous organisms, and fungivores also showed lower abundances at high altitude than at low altitude. However, the altitude gradient was less pronounced than in summer. Nevertheless, during winter, there was a treatment response for all trophic levels, with higher abundances in the OTCs, except for the detritivores, which showed no significant differences between altitude and treatment.

#### 3.1.2. Ecological Traits

With respect to ecological traits, during summer, low and high dispersers follow opposite trends, with a certain decrease along the altitudinal gradient for low dispersers and, inversely, an increase towards higher altitudes for high dispersers. During winter, high-dispersing arthropods also increase with altitude, but low-dispersing arthropods show no differences between fields. For both seasons, the OTCs showed a higher number of low-dispersal arthropods, while for the high-dispersal arthropods, the OTCs showed a lower number of individuals than the control plots. 

During the summer, the low-elevation field had the smallest body size community, while the medium-elevation field had the largest body size community. During winter, the arthropod body size increased with altitude, with larger arthropods at the highest altitude. Although there appears to have been a difference in body size between treatments in the high-altitude field during the summer, no significant differences were found (Figure 3 and Table A1).

In summer and winter, the diurnal arthropods decreased with altitude, while the nocturnal arthropods followed the opposite trend. In summer, the twilight arthropods decreased with altitude and in winter, they showed a hump shape along the altitude gradient with higher abundances at middle elevation. There were also fewer twilight and diurnal arthropods in the OTCs in winter.

#### 3.1.3. Feeding Behaviors

In terms of feeding behavior (Figure 3), chewing/cutting and piercing/sucking arthropods are less abundant at high altitude and show a slight decrease along the altitudinal gradient for both seasons. However, arthropods feeding by external digestion and sucking (e.g., spiders) are more abundant at the highest altitudes (Table A1 and Table A2).

### 3.2. Winter and Summer Beetle Trait Analysis

#### 3.2.1. Guilds

**Figure 4:**
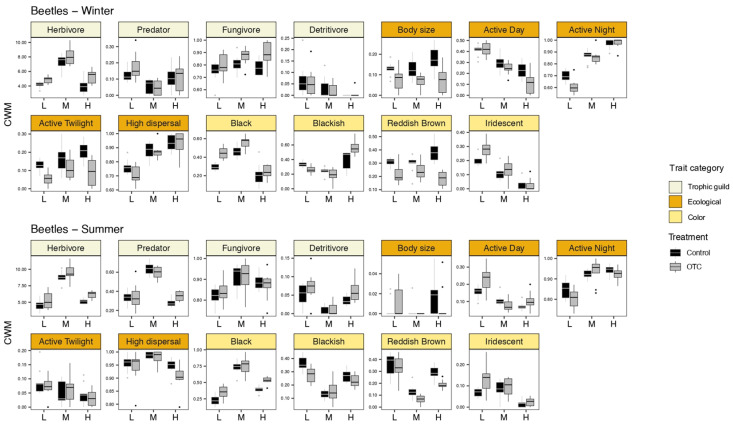
Boxplots highlighting the effect of the treatment (control vs. OTCs) on different traits along the elevation gradient considering only beetles during the winter and the summer. On *y*-axis are the value of the community weighted means (CWM) and on *x*-axis is the elevation factor: low (L), middle (M), and high (H) elevation. Black box plots indicate the control plots, while gray box plots represent the OTCs. ANOVA and post hoc pairwise comparisons results are available in Table A3 and Table A4.

In terms of guilds, there was no clear pattern across all the guilds. Herbivores follow a hump shape along the altitudinal gradient in summer and a U shape in winter. Thus, the middle altitude field shows a large seasonal variation in herbivore beetles (Figure 4).

The predators appeared to be homogeneous at different altitudes, with no major changes along the altitudinal gradient. However, in winter, more predators were observed inside the OTCs.

Fungivores do not show major changes between different altitudes and seasons. 

The detritivores were more abundant during the winter, perhaps due to the higher humidity and availability of decaying matter. In addition, fewer detritivores were observed inside the OTCs during winter.

#### 3.2.2. Ecological Traits

The beetle body sizes showed a hump shape along the altitudinal gradient, with larger species at middle altitude in both seasons. Larger beetles were also found in the OTCs during summer (Figure 4). 

The number of diurnal beetles decreased along the altitudinal gradient in both seasons. In winter, fewer diurnal beetles were observed inside the OTCs.

The number of nocturnal beetles tended to increase with altitude, in addition to the fact that fewer nocturnal beetles were present inside the OTCs during winter.

The crepuscular beetles showed an opposite pattern between winter and summer. They decreased along the altitudinal gradient in summer and increased in winter.

Finally, the high-dispersal beetles showed a hump shape along the altitudinal gradient in summer, but a clear increase with altitude in winter.

#### 3.2.3. Color and Iridescence

During the summer, the beetles with orange and red shades (blackish and reddish brown) appeared to be less abundant at middle altitude and showed a U-shape along the altitudinal gradient. The highest abundance was found at low elevation (Figure 4).

Contrastingly, the black beetles were significantly more abundant at mid-altitude in both seasons, showing a hump shape along the altitudinal gradient. The lowest abundances of black beetles were observed at low altitudes.

Differences between treatments were observed for the black and reddish-brown beetles in both seasons. More black beetles were observed in the OTCs, while more reddish-brown beetles were observed in control plots.

Finally, the iridescent beetles showed a clear and significant gradient along the altitudes and during both winter and summer. More iridescent beetles were observed at low altitudes, while the lowest number of iridescent beetles was found at high altitudes. During winter, differences between treatments were observed, with more iridescent beetles inside the OTCs, especially at low elevation.

## 4. Discussion

The analysis of the functional traits across the three elevations and temperature treatments provides valuable insights into the effects of climate and environmental change on arthropod communities. The results of this study also highlight the complex and diverse responses of arthropods to short-term climate change, with distinct patterns emerging across different functional traits and seasonal conditions.

### 4.1. Influence of Elevational Gradient and Seasonality

#### 4.1.1. Guilds

The elevational gradient significantly influenced the arthropod and beetle communities, with noticeable variations between seasons. For all the arthropods, during the summer, guilds such as herbivores, coprophagous organisms, detritivores, and fungivores showed lower abundances at high elevations. This supports previous findings that arthropod diversity and abundance tend to decrease with elevation [51,52], potentially due to the optimal balance between temperature, precipitation, and other environmental conditions [53,54]. However, the patterns were not uniform across all the guilds, with the predators showing more homogeneous distributions along the gradient, albeit still with a peak at middle elevation. This confirms the finding in previous studies that different trends in the response of arthropod abundance along the elevational gradient depended on the level of taxonomic and functional resolution [53]. In a recent elevational study on Terceira Island native forest habitats, mixed results were also obtained, with the total species richness of all arthropods, Coleoptera, and Psocodea showing a monotonic decrease with elevation, but peaking at mid-high elevation for Araneae and endemic species [55].

During the winter, the guild analyzed with all the arthropods showed mostly differences between treatments, which will be discussed in the following section.

The beetles during the summer, herbivores, and predators also showed a peak at middle elevation, agreeing with [53,54]. The numbers of fungivores and detritivore beetles were very low during summer, probably due to the fact that their distribution is closely linked to the availability of organic matter and litter quality. However, litter quality depends mostly on water availability and temperature [56]. The lack of litter quality in summer explains the low abundances of those guilds. Detritivore arthropods play a crucial role in the decomposition of dead organic matter, including plant material and animal remains [57]. In our experiment, most of the detritivores were not strictly decomposers of the organic matter of either animal or plant origin, but of both. Thus, during the summer, the drop in their abundance was mainly due to the quasi-absence of litter, which represented a considerable part of the diet of our detritivores. During the winter, these organisms often exhibit increased abundance, potentially due to the higher humidity and availability of decaying matter [58]. Indeed, detritivore beetles were more abundant during the winter, which aligns with findings that cooler temperatures often slow down decomposition rates, leading to a prolonged availability of decaying matter [59,60]. The higher humidity during winter months may further support the activity and abundance of detritivores, as moisture plays a crucial role in maintaining their physiological functions [61].

#### 4.1.2. Ecological Traits 

Another particularly interesting finding was the varying responses of arthropods with different dispersal abilities along the elevation gradient. During the summer, low-dispersal arthropods tended to decrease in abundance at higher elevations, while high-dispersal arthropods showed the opposite trend, becoming more abundant at higher elevations. This trend suggests that the ability to move more widely provides an advantage for some arthropods and beetles, as they track shifting climate conditions, a pattern observed in various ecosystems for arthropods and other taxa [62,63]. These findings highlight the critical need to consider dispersal capabilities when examining the ecological impacts of climate change on arthropod communities. 

One more key result was the shift in the body size distribution of the arthropod community along the elevation gradient, with larger-bodied species becoming more prevalent at higher elevations, particularly during the winter season. This finding aligns with previous research demonstrating that larger-bodied arthropods often succeed in colder, higher-elevation environments, probably due to metabolic and thermoregulatory adaptations [42,64,65]. The prevalence of larger-bodied species at higher elevations during winter may reflect their ability to better withstand harsher conditions, while smaller species may struggle to maintain their energetic requirements

However, the beetles exhibited distinct ecological traits along the elevation gradient. Our data show that two of the largest beetle species were more abundant at the middle elevation than at the low or high elevations, e.g., the staphylinid, *Ocypus olens* (Müller, 1764), and the ground Beetle, *Calosoma olivieri* Dejean, 1831. Therefore, contrary to expectations, our results showed that larger beetle species tend to peak at middle elevations. This is likely to be due to the favorable conditions for growth and development found at these levels for grassland arthropods, where resource availability and environmental conditions are well-balanced. Arthropod abundance generally peaks at higher elevations on gradients with a very dry base and a sharp increase in precipitation with elevation [66]. However, the prolonged developmental time required by larger insects can be disadvantageous at higher elevations due to shorter active seasons [67]. Therefore, climatic conditions pose a significant obstacle to their optimal development and may limit their distribution along elevational gradients [68]. However, these patterns can vary, since some of the most iconic large Azorean endemic forest-adapted ground-beetles are restricted to high-elevation, hyper-humid forests [69].

The daily activity patterns of all the arthropods and beetles showed that the crepuscular and diurnal arthropods decreased with elevation, while the nocturnal arthropods increased, a trend that was consistent across both seasons. The altered patterns of activity across the diurnal, nocturnal and crepuscular arthropods along the elevational gradient and between seasons suggests that climate change could disrupt important temporal niche partitioning within arthropod communities. Furthermore, diel activity may influence arthropod responses to increased temperatures, with nocturnal species potentially being less affected by daylight heat compared to diurnal species. This is because nocturnal arthropods remain hidden from direct solar exposure and extreme temperatures [70]. However, our experiment showed opposite results to these findings, with more diurnal activity at lower elevations and more nocturnal activity at higher elevations. Species-specific responses and the complex interplay of environmental factors, including food availability and predation pressure, also influence diel activity [71], and could explain the results of our experiment. 

This shift in activity patterns may also be due to the thermal constraints imposed by elevation (harsher conditions and colder periods), which can influence the temporal niche partitioning within arthropod communities. As Lobo (2024) [72] states, “hotter is not better”, and some species of beetle with specific metabolic mechanisms are able to maintain their nocturnal activity under cold temperatures. Different metabolic mechanisms can make it difficult for diurnal beetles to remain active at cooler temperatures. The crepuscular beetles exhibited opposite patterns between winter and summer, decreasing in summer and increasing in winter, possibly due to shifts in temperature and resource availability that influenced their activity periods.

This highlights the complex ways in which climate change can impact the structure and dynamics of arthropod communities, with cascading effects on ecosystem function.

#### 4.1.3. Feeding Behaviors

The feeding behaviors were notably influenced by elevation, with the chewing/cutting and piercing/sucking arthropods decreasing at higher elevations, while those utilizing external digestion (e.g., spiders) increased. This may reflect adaptations to resource availability and climatic conditions at different elevations. Conversely, predators and scavengers that rely on external digestion, such as spiders (mostly linyphiids in the current study), may have found a higher and more consistent grass cover throughout the year at the high elevation in our experiment, providing a more favorable ecosystem, as shown by Borges and Brown (2001 & 2004) [73,74]. Our data also revealed a high number of harvestmen from the Leiobunidae family, which also rely on external digestion, at high elevation. Their high number could have been due to the higher moisture levels that they tend to prefer [75], which naturally occurs at this elevation. These adaptations highlight the complex interplay between environmental conditions and arthropod feeding strategies across altitudinal gradients.

#### 4.1.4. Beetle Color and Iridescence

The results indicate the significant presence of iridescent beetles along the altitudinal gradient, with more iridescent beetles observed at lower elevations compared to higher elevations. This pattern suggests that iridescence may be an adaptation to cope with the higher temperatures typically found at lower elevations and emphasizes the role of iridescence in thermoregulation. Indeed, iridescence in beetles can serve various ecological functions, including camouflage, signaling, and thermoregulation [76,77,78]. In fact, iridescent surfaces reflect a significant portion of incident light, which can help regulate body temperature by reducing heat absorption. Conversely, at higher elevations, where temperatures are generally cooler, the advantage of iridescence in thermoregulation might be less pronounced, resulting in fewer iridescent beetles [79]. It is noteworthy that all Azorean endemic beetles found in high-elevation, hyper-humid forests (at approximately 500 m to 1000 m above sea level), although not part of this study, are typically non-iridescent and mostly blackish. In our study, we did not collect endemic Azorean beetles, since their habitat is at higher elevation and the Azorean intensive pastures are mostly composed of introduced species and very few native ones. Therefore, it would be interesting in the future to study the coloration of native and endemic beetles in more detail to see whether their response follows the same trend as that of introduced beetles. The observed coloration trends along the elevational gradient in our experiment reveal significant ecological and adaptive patterns in beetle populations. During the summer, the beetles with orange and red nuances, such as blackish and reddish-brown beetles, exhibited a U-shaped distribution, with the highest abundances at low elevations. This pattern suggests that these colorations may be more effective for thermoregulation and camouflage in the warmer environments found at lower elevations [80,81,82]. Conversely, the black beetles displayed a hump-shaped distribution, being more abundant at middle elevations during both seasons. The adaptive significance of melanism in black beetles, particularly for absorbing heat in cooler environments, might explain their success in these mid-altitudinal zones [83].

### 4.2. Effects of Increased Temperature (OTC Experiment)

#### 4.2.1. Guilds

The increased temperature of the OTCs played a crucial role in the arthropod and beetle traits observed, especially during winter. The higher abundances of herbivores, predators, coprophagous organisms, and fungivores within the OTCs during winter suggest that increased temperatures may alleviate some environmental stressors, leading to increased activity and survival. Vasseur et al. [84] agree that an increase in temperature has these effects on ectotherms. However, they caution that it is important to consider the effect of the temperature mean and variance, as their model predicts that mid-latitude species will be most susceptible to large declines in performance under a future climate scenario when these two factors are considered. Furthermore, higher temperatures in OTCs during winter may reduce the energy costs associated with thermoregulation (for warming), allowing more resources to be allocated to foraging, mating, and development [85,86]. Such effects are known to be particularly pronounced in winter for insects, when thermal stress is typically higher, and the relative benefits of small temperature increases can be substantial [87].

The detritivores, arthropods that feed on decaying matter, did not show significant differences between the treatments. The rate of detrital decomposition depends on the quality of the detritus and the physical environment, including factors such as temperature and water availability [56]. In the Azores, precipitation is highest from November to January, and it varies considerably within each month, depending on elevation and longitude. Humidity is generally high, with median relative humidity (RH) values around 80%, and the cooler months are typically January and February [88], with temperatures above 0 °C in our experiment. Therefore, detritivores may be particularly dependent on the availability of decaying matter and less affected by temperature changes than other guilds.

For the beetles, increased temperatures impacted most of the functional traits studied, with stronger effects also observed during winter. This suggests that beetles may be more sensitive to temperature changes during colder months, potentially due to metabolic and physiological constraints. Ectotherms, such as beetles, rely on external sources of heat to regulate their body temperature, and their metabolic rates are strongly influenced by ambient temperatures [85,86]. At lower temperatures, metabolic rates are typically slower, potentially limiting activity levels and overall fitness [89]. Therefore, even small increases in temperature during winter could have a disproportionately large impact on beetle physiology and behavior, aligning with findings from other studies demonstrating the vulnerability of ectotherms to temperature changes, particularly during colder periods [87].

During the winter season, the detritivore arthropod abundance may have been lower within the OTCs compared to the natural control plots due to several factors. Temperature variation within OTCs can disrupt the stable microhabitat that detritivores rely on [90]. While OTCs are designed to elevate overall temperatures, the enclosed environment may lead to greater daily and seasonal fluctuations in temperature compared to the surrounding natural plots [91]. This could have adversely affected the physiology and behavior of these moisture-dependent organisms, making the environment less suitable for their survival and reproduction. In addition to temperature, OTCs may also alter moisture levels within leaf litter. By trapping heat, OTCs can potentially dry out the organic substrate, reducing the humidity levels that detritivores require [92]. Alternatively, the OTCs may have created a more condensed environment, leading to excessive moisture, which is also detrimental to detritivore communities. Furthermore, the physical presence of the OTC structures themselves could have interfered with the natural accumulation and distribution of organic matter, which serves as the primary food source for detritivore arthropods. This disruption of the microhabitat could have limited the availability of resources and led to a decreased abundance of these decomposer organisms within the OTCs compared to the control plots.

#### 4.2.2. Ecological Traits

Interestingly, while the beetle body size peaked at middle elevations in both summer and winter, a larger average body size was observed within the OTCs during the summer. This contradicts the general trend of smaller body sizes in ectotherms under warmer temperatures, as suggested by [42,93]. This discrepancy could be attributed to species-specific responses to temperature, altered resource availability within the OTCs, or interactions with other environmental factors not explored in this study. For instance, increased temperatures might have led to a greater quality and amount of food for certain beetle species [94]. 

The reduced activity of the nocturnal beetles, as well as the reduced activity of the diurnal and crepuscular arthropods and beetles within the OTC treatments during the winter, may indicate a means of avoiding thermal stress, as suggested by Thiele [70]. However, this result needs to be considered with caution, as in our case, the temperature within the OTCs during the winter was warmer than in the control plots, but not extreme. Therefore, it should be considered whether other factors, such as food availability, could have been responsible for the observed effect, and not only thermal stress, which should be low at this time of year. Pawar et al. (2024) [95] also highlighted that thermal adaptation in arthropods is constrained by the temperature of peak performance for key life-history traits, which could also explain our results.

More low-dispersal arthropods were found in the OTCs than in the control plots. The opposite was found for the high-dispersal arthropods, which were more abundant in the control plots than in the OTCs. Thus, the high dispersers may have left the OTCs when the abiotic conditions became unsuitable, with only low dispersers remaining. This pattern is consistent with the finding that as climate change occurs, high dispersers are the first to seek out better abiotic conditions [96,97]. 

During the summer, larger beetles were more prevalent in the OTCs, potentially indicating that increased temperatures within OTCs favor the presence of some larger beetle species. This finding opposes the prevailing trend in ectotherms, which tend to have smaller body sizes at warmer temperatures [42], suggesting species-specific responses to temperature or altered resource dynamics within OTCs.

#### 4.2.3. Feeding Behavior

The study found that the feeding behavior of arthropods, including piercing, sucking, chewing, cutting, and external digestion, was not impacted by increased temperatures within the OTCs. Although mouthparts are unique to each arthropod kingsofamily, this finding is also consistent with several studies suggesting that while temperature can affect metabolic rates and activity levels, the abundance of different arthropod feeding strategies often remains constant across temperature variations [98,99,100].

#### 4.2.4. Beetle Color and Iridescence

The data showed an increased number of iridescent beetles inside the OTCs at lower elevations during winter, suggesting that elevated temperatures enhance the prevalence of iridescence. The increased presence of iridescent beetles inside the OTCs implies that iridescence may provide a thermoregulatory benefit under higher temperatures, aiding in heat reflection and reducing thermal stress [76,79]. This ability to reflect sunlight and maintain lower body temperatures would be particularly advantageous during warmer periods, supporting the survival and activity of iridescent beetles [101]. 

The higher abundance of black beetles in the OTCs during winter suggests potential thermal benefits associated with darker coloration. Darker colors can absorb more heat, providing significant thermal advantages in colder environments [80,81]. This adaptation probably enhanced the survival and activity of the black beetles during the winter months, when ambient temperatures are lower. The observation of fewer black beetles in the control plots compared to the OTCs supports the hypothesis that the increased temperatures within the OTCs would create more favorable conditions for these beetles in winter. Such findings align with the theory that melanism confers thermal benefits by facilitating heat absorption, which is crucial for ectotherms to maintain metabolic processes in cooler climates [83]. The reddish-brown coloration might offer a balance between camouflage and thermal regulation, providing sufficient heat absorption without the risk of overheating in moderate environments [82,102,103].

These findings contribute to our understanding of how beetles adapt to varying microclimatic conditions and offer a basis for further research on the ecological functions of beetle coloration and iridescence.

## 5. Conclusions

Overall, this study highlights the complex interplay between elevation, temperature, and season in shaping arthropod and beetle communities. The observed patterns emphasize the need to consider multiple environmental factors when assessing the impact of climate change on biodiversity. The functional traits approach unveiled more patterns among the studied arthropod communities in response to climatic changes than the taxonomical approach previously applied by Wallon et al. (2023) [30]. Other studies comparing the taxonomical and the functional approach showed the importance of considering functional traits in analyzing arthropod communities and agreed that the functional approach enhances knowledge of the processes underlying patterns of diversity in complex ecological communities involving multiple trophic levels [104,105,106]. However, analyses including functional traits appear to be more complex to interpret due to various factors and interactions [107]. 

Future research should further explore the underlying mechanisms driving these patterns and their implications for ecosystem functioning and resilience. The results of this study contribute to this growing body of knowledge and highlight the importance of examining arthropod responses at the community level and considering functional traits, rather than focusing solely on individual species.

In conclusion, the observed effects of increasing temperature and elevational gradients on the functional traits of arthropods and beetles highlight the complex and functional-group-specific nature of thermal responses within these communities.

## Figures and Tables

**Table 1:** Selection, definition, and ecological relevance of the functional traits used in the study.

Traits Category	Traits	Data Type	Attributes (Abbreviations)	Definition	Ecological Relevance
Guild	Type of food	Multi-choice nominal	Plants (FoodPl); animals (FoodAni); fungi (FoodFg); detritus (FoodDet); coprophagous organisms (FoodCopro)	Each guild is categorized according to the primary food consumed by the species during their adult stages, except for Lepidoptera, for which the classification is based on the feeding habits of the larvae.	Under different climatic conditions, the natural resources available for arthropod feeding may vary. This can affect arthropod feeding guilds and lead to different arthropod communities.
Feeding behavior	Mode of ingestion	Nominal	Chewing and cutting (IngCC); piercing and sucking (IngPS); external digestion and sucking (IngEDS)	Depends on the type of mouthpart and, thus, on the way in which arthropods feed. For Lepidoptera, the classification is based on the mouthparts of the larvae.	Determines feeding strategies and diet specialization, influencing nutrient cycling and energy flow within ecosystems.
Ecological	Body size	Continuous	Standardized body size (bodysizeStand); body size (bodysize)	Defined as species mean body length.	Species’ body size is linked to their metabolism, which influences their adaptability and ecological niches.
Ecological	Dispersal ability	Nominal	Low dispersal ability (Ldisp); High dispersal ability (Hdisp)	Dispersal ability (high or low) is attributed based on physical characteristics of the animal. Presence or absence of wings and ballooning capacity for spiders.	Animals with high dispersal ability will tend to respond more quickly to unsuitable abiotic conditions in order to find a more suitable environment. In contrast, animals with low dispersal ability will have more difficulty in migrating to better conditions.
Ecological	Diel activity	Multi-choice nominal	Day (ActDay); Night (ActNig); Twilight (ActTw)	Refers to the main period of activity of the species during the day.	Diel activity shapes how species interact with their environment and each other, and also impacts how arthropods respond to heat. Nocturnal species are less affected by daytime temperatures, as they avoid direct sunlight and extreme heat by remaining hidden.
Coloration	Color of beetle cuticle	Nominal	Black (Color_Black); blackish (Color_Blackish); reddish-brown (Color_ReddishBrown)	Refers to the main nuance observed on the cuticle. Black refers to a black cuticle. Blackish refers to a main black tendency with a reddish/orange color. Reddish-brown refers to a main tendency orange/brown.	Color can serve several functions, including signaling, mating, camouflage, and thermoregulation. For this reason, beetle color should provide information about the heat tolerance of the species.
Coloration	Presence of iridescence on beetle cuticle	Nominal	Iridescence (IridescentColor)	Refers to the presence or absence of iridescence on the beetle cuticle.	The presence of iridescence helps thermoregulation by reducing solar absorption and limiting risks of overheating.

## Data Availability

Data used for this publication are available at: Wallon, S.; Elias, R.B.; Borges, P.A.V. (2023). Monitoring grassland arthropods in an in situ climate change experiment (Terceira, Azores, Portugal). Version 1.5. Universidade dos Açores. Sampling event dataset, https://doi.org/10.15468/u2xh5g accessed on 19 July 2024, via GBIF.org.

## Supplementary Materials

The following supporting information can be downloaded at: https://www.mdpi.com/article/10.3390/insects15090677/s1. Table S1: Species collected over the winter and the summer at all sites studied; Table S2: Beetle species collected over the winter and the summer at all sites studied.

## Appendix A

**Table A1:** Results of the ANOVA performed to investigate the effects of the elevation and the treatment on functional traits of all arthropods, including the interaction terms (Elev:Treatment) on each CWM variable. Adjusted *p* values are also presented. Significant results are marked in bold. Traits presented are Herbivore; Predator; Fungivore; Detritivore, Coprophagous; External digestion and sucking (IngestEDS); Chewing and cutting (CC); Piercing and sucking (PS); Standardized body size (Body size) in (mm); High dispersal ability (Hdisp) and low dispersal ability (Ldisp); Diurnal arthropods (ActDay); Nocturnal arthropods (ActNig); and arthropods active during twilight (ActTwi).

Season	Trait	Factor	Df	Sum Sq.	Mean Sq.	F Value	Pr(>F)	*p* Adjust
Summer	Herbivore	Elevation	2	0.847	0.424	212.617	**<0.001**	**<0.001**
		Treatment	1	0.001	0.001	0.360	0.551	0.605
		Elev:Treatment	2	0.029	0.015	7.359	**0.001**	**0.004**
		Residuals	54	0.108	0.002			
Winter	Herbivore	Elevation	2	0.147	0.073	22.520	**<0.001**	**<0.001**
		Treatment	1	0.075	0.075	23.106	**<0.001**	**<0.001**
		Elev:Treatment	2	0.006	0.003	0.981	0.382	0.477
		Residuals	54	0.176	0.003			
Summer	Predator	Elevation	2	0.029	0.014	8.667	**0.001**	**0.002**
		Treatment	1	0.000	0.000	0.235	0.629	0.659
		Elev:Treatment	2	0.011	0.005	3.227	**0.047**	0.082
		Residuals	54	0.089	0.002			
Winter	Predator	Elevation	2	0.079	0.040	12.293	**<0.001**	**<0.001**
		Treatment	1	0.031	0.031	9.772	**0.003**	**0.008**
		Elev:Treatment	2	0.003	0.001	0.400	0.672	0.756
		Residuals	54	0.174	0.003			
Summer	Fungivore	Elevation	2	0.204	0.102	97.826	**<0.001**	**<0.001**
		Treatment	1	0.008	0.008	8.028	**0.006**	**0.014**
		Elev:Treatment	2	0.004	0.002	1.87	0.164	0.217
		Residuals	54	0.056	0.001			
Winter	Fungivore	Elevation	2	0.135	0.068	28.955	**<0.001**	**<0.001**
		Treatment	1	0.018	0.018	7.510	**0.008**	**0.020**
		Elev:Treatment	2	0.002	0.001	0.339	0.714	0.765
		Residuals	54	0.126	0.002			
Summer	Detritivore	Elevation	2	0.140	0.070	53.575	**<0.001**	**<0.001**
		Treatment	1	0.014	0.014	10.820	**0.002**	**0.004**
		Elev:Treatment	2	0.006	0.003	2.318	0.108	0.147
		Residuals	54	0.070	0.001			
Winter	Detritivore	Elevation	2	0.003	0.001	0.516	0.600	0.711
		Treatment	1	0.000	0.000	0.138	0.712	0.765
		Elev:Treatment	2	0.003	0.002	0.595	0.555	0.675
		Residuals	54	0.146	0.003			
Summer	Coprophagous	Elevation	2	0.234	0.117	99.245	**<0.001**	**<0.001**
		Treatment	1	0.004	0.004	3.712	0.059	0.098
		Elev:Treatment	2	0.004	0.002	1.638	0.204	0.262
		Residuals	54	0.064	0.001			
Winter	Coprophagous	Elevation	2	0.083	0.041	20.037	**<0.001**	**<0.001**
		Treatment	1	0.022	0.022	10.808	**0.002**	**0.005**
		Elev:Treatment	2	0.000	0.000	0.087	**0.917**	0.923
		Residuals	54	0.111	0.002			
Summer	IngestEDS	Elevation	2	0.212	0.106	81.22	**<0.001**	**<0.001**
		Treatment	1	0.004	0.004	3.212	**0.079**	0.116
		Elev:Treatment	2	0.014	0.007	5.306	**0.008**	**0.016**
		Residuals	54	0.070	0.001			
Winter	IngestEDS	Elevation	2	0.152	0.076	37.49	**<0.001**	**<0.001**
		Treatment	1	0.008	0.008	3.999	0.051	0.099
		Elev:Treatment	2	0.004	0.002	1.098	0.341	0.477
		Residuals	54	0.110	0.002			
Summer	IngestCC	Elevation	2	0.147	0.074	46.258	**<0.001**	**<0.001**
		Treatment	1	0.001	0.001	0.572	0.453	0.536
		Elev:Treatment	2	0.023	0.012	7.307	**0.002**	**0.004**
		Residuals	54	0.086	0.002			
Winter	IngestCC	Elevation	2	0.115	0.057	29.110	**<0.001**	**<0.001**
		Treatment	1	0.012	0.012	5.907	**0.018**	**0.041**
		Elev:Treatment	2	0.005	0.003	1.360	0.265	0.426
		Residuals	54	0.107	0.002			
Summer	IngestPS	Elevation	2	0.013	0.006	23.312	**<0.001**	**<0.001**
		Treatment	1	0.001	0.001	3.653	0.061	0.098
		Elev:Treatment	2	0.002	0.001	3.530	**0.036**	0.065
		Residuals	54	0.015	0.000			
Winter	IngestPS	Elevation	2	0.004	0.002	10.916	**<0.001**	**<0.001**
		Treatment	1	0.001	0.001	4.348	**0.042**	0.086
		Elev:Treatment	2	0.000	0.000	0.186	0.831	0.869
		Residuals	54	0.009	0.000			
Summer	Body size	Elevation	2	2350.500	1175.300	61.207	**<0.001**	**<0.001**
		Treatment	1	61.200	61.200	3.187	0.080	0.116
		Elev:Treatment	2	102.000	51.000	2.657	0.079	0.116
		Residuals	54	1036.900	19.200			
Winter	Body size	Elevation	2	809.300	404.600	21.583	**<0.001**	**<0.001**
		Treatment	1	15.000	15.000	0.801	0.375	0.477
		Elev:Treatment	2	66.700	33.300	1.778	0.179	0.309
		Residuals	54	1012.400	18.700			
Summer	Hdisp	Elevation	2	0.544	0.272	213.338	**<0.001**	**<0.001**
		Treatment	1	0.031	0.031	24.141	**<0.001**	**<0.001**
		Elev:Treatment	2	0.013	0.006	4.923	**0.011**	**0.021**
		Residuals	54	0.069	0.001			
Winter	Hdisp	Elevation	2	0.354	0.177	46.911	**<0.001**	**<0.001**
		Treatment	1	0.049	0.049	13.067	**<0.001**	**0.002**
		Elev:Treatment	2	0.004	0.002	0.457	0.636	0.733
		Residuals	54	0.204	0.004			
Summer	Ldisp	Elevation	2	0.018	0.009	10.41	**<0.001**	**<0.001**
		Treatment	1	0.016	0.016	18.81	**<0.001**	**<0.001**
		Elev:Treatment	2	0.008	0.004	4.41	**<0.001**	**0.032**
		Residuals	54	0.047	0.001			
Winter	Ldisp	Elevation	2	0.011	0.005	2.207	0.120	0.225
		Treatment	1	0.022	0.022	9.226	**0.004**	**0.009**
		Elev:Treatment	2	0.000	0.000	0.08	0.923	0.923
		Residuals	54	0.131	0.002			
Summer	ActivDay	Elevation	2	0.492	0.246	159.662	**<0.001**	**<0.001**
		Treatment	1	0.001	0.001	0.794	0.377	0.471
		Elev:Treatment	2	0.008	0.004	2.578	0.085	0.120
		Residuals	54	0.083	0.002			
Winter	ActivDay	Elevation	2	0.458	0.229	74.253	**<0.001**	**<0.001**
		Treatment	1	0.018	0.018	5.734	**0.020**	**0.043**
		Elev:Treatment	2	0.011	0.006	1.826	0.171	0.308
		Residuals	54	0.167	0.003			
Summer	ActivNight	Elevation	2	0.014	0.007	11.8	**<0.001**	**<0.001**
		Treatment	1	0.000	0.000	0.027	0.869	0.869
		Elev:Treatment	2	0.001	0.000	0.501	0.609	0.652
		Residuals	54	0.032	0.001			
Winter	ActivNight	Elevation	2	0.357	0.178	161.993	**<0.001**	**<0.001**
		Treatment	1	0.002	0.002	1.59	0.213	0.355
		Elev:Treatment	2	0.002	0.001	1.066	0.352	0.477
		Residuals	54	0.060	0.001			
Summer	ActivTwi	Elevation	2	0.396	0.198	121.950	**<0.001**	**<0.001**
		Treatment	1	0.000	0.000	0.213	0.646	0.661
		Elev:Treatment	2	0.003	0.001	0.767	0.470	0.542
		Residuals	54	0.088	0.002			
Winter	ActivTwi	Elevation	2	0.082	0.041	17.828	**<0.001**	**<0.001**
		Treatment	1	0.028	0.028	12.182	**0.001**	**0.003**
		Elev:Treatment	2	0.006	0.003	1.218	0.304	0.472
		Residuals	54	0.124	0.002			

**Table A2:** Results of the post hoc pairwise comparisons performed on the different traits of all arthropods when the ANOVA models were significant. Significant results are marked in bold. Traits presented are Herbivore; Predator; Fungivore; Detritivore, Coprophagous; External digestion and sucking (IngestEDS); Chewing and cutting (CC); Piercing and sucking (PS); Standardized body size (Body size) in (mm); High dispersal ability (Hdisp) and low dispersal ability (Ldisp); Diurnal arthropods (ActDay); Nocturnal arthropods (ActNig); and arthropods active during twilight (ActTwi). Factors presented are the elevation, the treatment, and their interaction (Elev:Treatment). Low-elevation field (LOW); Middle-elevation field (MID); High-elevation field (HIGH); Control plots at low elevation (LOW C.); OTCs at low elevation (LOW OTC); Control plots at middle elevation (MID C.); OTCs at middle elevation (MID OTC); Control plots at high elevation (HIGH C.); OTCs at high elevation (HIGH OTC). See corresponding box plot in the main text in Figure 3.

Season	Trait	Factor	Pairwise Comparisons	Estimate	SE	df	t.Ratio	*p* Value	Trend
Summer	Herbivore	Elevation	LOW–MID	−0.045	0.014	54	−3.196	**0.007**	LOW < MID
			LOW–HIGH	0.227	0.014	54	16.045	**<0.001**	LOW > HIGH
			MID–HIGH	0.272	0.014	54	19.241	**<0.001**	MID > HIGH
Summer	Herbivore	Elev:Treatment	LOW C.–MID C.	−0.075	0.020	54	−3.763	**0.005**	LOW C. < MID C.
			LOW C.–HIGH C.	0.251	0.020	54	12.550	**<0.001**	LOW C. > HIGH C.
			LOW C.–LOW OTC	−0.011	0.020	54	−0.545	0.994	LOW C. = LOW OTC
			LOW C.–MID OTC	−0.026	0.020	54	−1.302	0.782	LOW C. = MID OTC
			LOW C.–HIGH OTC	0.192	0.020	54	9.596	**<0.001**	LOW C. > HIGH OTC
			MID C.–HIGH C.	0.326	0.020	54	16.313	**<0.001**	MID C. > HIGH C.
			MID C.–LOW OTC	0.064	0.020	54	3.218	**0.025**	MID C. > LOW OTC
			MID C.–MID OTC	0.049	0.020	54	2.460	0.155	MID C. = MID OTC
			MID C.–HIGH OTC	0.267	0.020	54	13.358	**<0.001**	MID C. > HIGH OTC
			HIGH C.–LOW OTC	−0.261	0.020	54	−13.095	**<0.001**	HIGH C. < LOW OTC
			HIGH C.–MID OTC	−0.277	0.020	54	−13.852	**<0.001**	HIGH C. < MID OTC
			HIGH C.–HIGH OTC	−0.059	0.020	54	−2.954	0.050	HIGH C. = HIGH OTC
			LOW OTC–MID OTC	−0.015	0.020	54	−0.757	0.973	LOW OTC = MID OTC
			LOW OTC–HIGH OTC	0.202	0.020	54	10.141	**<0.001**	LOW OTC > HIGH OTC
			MID OTC–HIGH OTC	0.218	0.020	54	10.898	**<0.001**	MID OTC > HIGH OTC
Winter	Herbivore	Elevation	LOW–MID	0.096	0.018	54	5.301	**<0.001**	LOW > MID
			LOW–HIGH	0.112	0.018	54	6.215	**<0.001**	LOW > HIGH
			MID–HIGH	0.017	0.018	54	0.914	0.634	MID = HIGH
Winter	Herbivore	Treatment	Control–OTC	−0.071	0.015	54	−4.807	**<0.001**	C < OTC
Summer	Predator	Elevation	LOW–MID	−0.052	0.013	54	−4.026	**<0.001**	LOW < MID
			LOW–HIGH	−0.014	0.013	54	−1.094	0.522	LOW = HIGH
			MID–HIGH	0.038	0.013	54	2.932	**0.013**	MID > HIGH
Summer	Predator	Elev:Treatment	LOW C.–MID C.	−0.070	0.018	54	−3.841	**0.004**	LOW C. < MID C.
			LOW C.–HIGH C.	−0.047	0.018	54	−2.566	0.124	LOW C. = HIGH C.
			LOW C.–LOW OTC	−0.039	0.018	54	−2.138	0.284	LOW C. = LOW OTC
			LOW C.–MID OTC	−0.072	0.018	54	−3.991	**0.003**	LOW C. < MID OTC
			LOW C.–HIGH OTC	−0.020	0.018	54	−1.118	0.872	LOW C. = HIGH OTC
			MID C.–HIGH C.	0.023	0.018	54	1.274	0.797	MID C. = HIGH C.
			MID C.–LOW OTC	0.031	0.018	54	1.703	0.536	MID C. = LOW OTC
			MID C.–MID OTC	−0.003	0.018	54	−0.150	1.000	MID C. = MID OTC
			MID C.–HIGH OTC	0.049	0.018	54	2.722	0.087	MID C. = HIGH OTC
			HIGH C.–LOW OTC	0.008	0.018	54	0.428	0.998	HIGH C. = LOW OTC
			HIGH C.–MID OTC	−0.026	0.018	54	−1.425	0.712	HIGH C. = MID OTC
			HIGH C.–HIGH OTC	0.026	0.018	54	1.448	0.698	HIGH C. = HIGH OTC
			LOW OTC–MID OTC	−0.034	0.018	54	−1.853	0.442	LOW OTC = MID OTC
			LOW OTC–HIGH OTC	0.019	0.018	54	1.020	0.909	LOW OTC = HIGH OTC
			MID OTC–HIGH OTC	0.052	0.018	54	2.872	0.061	MID OTC = HIGH OTC
Winter	Predator	Elevation	LOW–MID	−0.085	0.018	54	−4.720	**<0.001**	LOW < MID
			LOW–HIGH	−0.019	0.018	54	−1.046	0.552	LOW = HIGH
			MID–HIGH	0.066	0.018	54	3.675	**0.002**	MID > HIGH
Winter	Predator	Treatment	Control–OTC	−0.046	0.015	54	−3.126	**0.003**	C < OTC
Summer	Fungivore	Elevation	LOW–MID	0.043	0.010	54	4.205	**<0.001**	LOW > MID
			LOW–HIGH	0.140	0.010	54	13.656	**<0.001**	LOW > HIGH
			MID–HIGH	0.097	0.010	54	9.450	**<0.001**	MID > HIGH
Summer	Fungivore	Treatment	Control–OTC	−0.024	0.008	54	−2.833	**0.007**	C < OTC
Winter	Fungivore	Elevation	LOW–MID	0.013	0.0153	54	0.852	0.673	LOW = MID
			LOW–HIGH	0.1066	0.0153	54	6.975	**<0.001**	LOW > HIGH
			MID–HIGH	0.0936	0.0153	54	6.123	**<0.001**	MID > HIGH
Winter	Fungivore	Treatment	Control–OTC	−0.034	0.0125	54	−2.741	**0.008**	C < OTC
Summer	Detritivore	Elevation	LOW–MID	0.0504	0.0114	54	4.411	**0.000**	LOW > MID
			LOW–HIGH	0.1178	0.0114	54	10.315	**<0.001**	LOW > HIGH
			MID–HIGH	0.0674	0.0114	54	5.904	**<0.001**	MID > HIGH
Summer	Detritivore	Treatment	Control–OTC	−0.031	0.009	54	−3.289	**0.002**	C < OTC
Summer	Coprophagous	Elevation	LOW–MID	0.015	0.011	54	1.381	0.358	LOW = MID
			LOW–HIGH	0.139	0.011	54	12.833	**<0.001**	LOW > HIGH
			MID–HIGH	0.124	0.011	54	11.452	**<0.001**	MID > HIGH
Winter	Coprophagous	Elevation	LOW–MID	−0.009	0.014	54	−0.608	0.816	LOW = MID
			LOW–HIGH	0.074	0.014	54	5.153	**<0.001**	LOW > HIGH
			MID–HIGH	0.083	0.014	54	5.761	**<0.001**	MID > HIGH
Winter	Coprophagous	Treatment	Control–OTC	−0.039	0.012	54	−3.288	**0.002**	C < OTC
Summer	IngestCC	Elevation	LOW–MID	−0.036	0.013	54	−2.857	0.016	LOW = MID
			LOW–HIGH	0.082	0.013	54	6.526	**<0.001**	LOW > HIGH
			MID–HIGH	0.118	0.013	54	9.382	**<0.001**	MID > HIGH
Summer	IngestCC	Elev:Treatment	LOW C.–MID C.	−0.069	0.018	54	−3.867	**0.004**	LOW C. < MID C.
			LOW C.–HIGH C.	0.096	0.018	54	5.400	**<0.001**	LOW C. > HIGH C.
			LOW C.–LOW OTC	−0.005	0.018	54	−0.271	1.000	LOW C. = LOW OTC
			LOW C.–MID OTC	−0.008	0.018	54	−0.444	0.998	LOW C. = MID OTC
			LOW C.–HIGH OTC	0.064	0.018	54	3.558	**0.010**	LOW C. > HIGH OTC
			MID C.–HIGH C.	0.165	0.018	54	9.267	**<0.001**	MID C. > HIGH C.
			MID C.–LOW OTC	0.064	0.018	54	3.596	**0.009**	MID C. > LOW OTC
			MID C.–MID OTC	0.061	0.018	54	3.423	**0.014**	MID C. > MID OTC
			MID C.–HIGH OTC	0.133	0.018	54	7.424	**<0.001**	MID C. > HIGH OTC
			HIGH C.–LOW OTC	−0.101	0.018	54	−5.671	**<0.001**	HIGH C. < LOW OTC
			HIGH C.–MID OTC	−0.104	0.018	54	−5.844	**<0.001**	HIGH C. < MID OTC
			HIGH C.–HIGH OTC	−0.033	0.018	54	−1.843	0.448	HIGH C. = HIGH OTC
			LOW OTC–MID OTC	−0.003	0.018	54	−0.173	1.000	LOW OTC = MID OTC
			LOW OTC–HIGH OTC	0.068	0.018	54	3.828	**0.004**	LOW OTC > HIGH OTC
			MID OTC–HIGH OTC	0.071	0.018	54	4.001	**0.003**	MID OTC > HIGH OTC
Winter	IngestCC	Elevation	LOW–MID	0.094	0.014	54	6.683	**<0.001**	LOW > MID
			LOW–HIGH	0.092	0.014	54	6.530	**<0.001**	LOW > HIGH
			MID–HIGH	−0.002	0.014	54	−0.153	0.987	MID = HIGH
Winter	IngestCC	Treatment	Control–OTC	0.028	0.012	54	2.431	**0.018**	C > OTC
Summer	IngestEDS	Elevation	LOW–MID	0.016	0.011	54	1.413	0.341	LOW = MID
			LOW–HIGH	−0.117	0.011	54	−10.263	**<0.001**	LOW < HIGH
			MID–HIGH	−0.133	0.011	54	−11.676	**<0.001**	MID < HIGH
Summer	IngestEDS	Treatment	Control–OTC	−0.017	0.009	54	−1.792	0.079	C = OTC
Summer	IngestEDS	Elev:Treatment	LOW C.–MID C.	0.038	0.016	54	2.328	0.201	LOW C. = MID C.
			LOW C.–HIGH C.	−0.133	0.016	54	−8.222	**<0.001**	LOW C. < HIGH C.
			LOW C.–LOW OTC	−0.013	0.016	54	−0.793	0.968	LOW C. = LOW OTC
			LOW C.–MID OTC	−0.018	0.016	54	−1.122	0.870	LOW C. = MID OTC
			LOW C.–HIGH OTC	−0.114	0.016	54	−7.084	**<0.001**	LOW C. < HIGH OTC
			MID C.–HIGH C.	−0.170	0.016	54	−10.550	**<0.001**	MID C. < HIGH C.
			MID C.–LOW OTC	−0.050	0.016	54	−3.121	**0.033**	MID C. < LOW OTC
			MID C.–MID OTC	−0.056	0.016	54	−3.450	**0.013**	MID C. < MID OTC
			MID C.–HIGH OTC	−0.152	0.016	54	−9.412	**<0.001**	MID C. < HIGH OTC
			HIGH C.–LOW OTC	0.120	0.016	54	7.430	**<0.001**	HIGH C. > LOW OTC
			HIGH C.–MID OTC	0.115	0.016	54	7.101	**<0.001**	HIGH C. > MID OTC
			HIGH C.–HIGH OTC	0.018	0.016	54	1.138	0.863	HIGH C. = HIGH OTC
			LOW OTC–MID OTC	−0.005	0.016	54	−0.329	1.000	LOW OTC = MID OTC
			LOW OTC–HIGH OTC	−0.102	0.016	54	−6.292	**<0.001**	LOW OTC < HIGH OTC
			MID OTC–HIGH OTC	−0.096	0.016	54	−5.962	**<0.001**	MID OTC < HIGH OTC
Winter	IngestEDS	Elevation	LOW–MID	−0.102	0.014	54	−7.175	**<0.001**	LOW < MID
			LOW–HIGH	−0.111	0.014	54	−7.785	**<0.001**	LOW < HIGH
			MID–HIGH	−0.009	0.014	54	−0.610	0.815	MID = HIGH
Summer	IngestPS	Elevation	LOW–MID	0.024	0.005	54	4.510	**<0.001**	A > B
			LOW–HIGH	0.035	0.005	54	6.695	**<0.001**	A > C
			MID–HIGH	0.011	0.005	54	2.185	0.083	B = C
Summer	IngestPS	Elev:Treatment	LOW C.–MID C.	0.036	0.007	54	4.912	**<0.001**	LOW C. > MID C.
			LOW C.–HIGH C.	0.036	0.007	54	4.947	**<0.001**	LOW C. > HIGH C.
			LOW C.–LOW OTC	0.018	0.007	54	2.394	0.177	LOW C. = LOW OTC
			LOW C.–MID OTC	0.028	0.007	54	3.860	**0.004**	LOW C. > MID OTC
			LOW C.–HIGH OTC	0.051	0.007	54	6.915	**<0.001**	LOW C. > HIGH OTC
			MID C.–HIGH C.	0.000	0.007	54	0.035	1.000	MID C. = HIGH C.
			MID C.–LOW OTC	−0.019	0.007	54	−2.518	0.137	MID C. = LOW OTC
			MID C.–MID OTC	−0.008	0.007	54	−1.052	0.898	MID C. = MID OTC
			MID C.–HIGH OTC	0.015	0.007	54	2.004	0.354	MID C. = HIGH OTC
			HIGH C.–LOW OTC	−0.019	0.007	54	−2.553	0.127	HIGH C. = LOW OTC
			HIGH C.–MID OTC	−0.008	0.007	54	−1.087	0.885	HIGH C. = MID OTC
			HIGH C.–HIGH OTC	0.015	0.007	54	1.969	0.373	HIGH C. = HIGH OTC
			LOW OTC–MID OTC	0.011	0.007	54	1.466	0.687	LOW OTC = MID OTC
			LOW OTC–HIGH OTC	0.033	0.007	54	4.522	**0.001**	LOW OTC > HIGH OTC
			MID OTC–HIGH OTC	0.023	0.007	54	3.056	**0.039**	MID OTC > HIGH OTC
Winter	IngestPS	Elevation	LOW–MID	0.012	0.004	54	2.931	**0.014**	A > B
			LOW–HIGH	0.019	0.004	54	4.617	**<0.001**	A > C
			MID–HIGH	0.007	0.004	54	1.686	0.220	B = C
Winter	IngestPS	Treatment	Control–OTC	−0.007	0.003	54	−2.085	**0.042**	C < OTC
Summer	Body size	Elevation	LOW–MID	−15.25	1.39	54	−11.004	**<0.001**	LOW < MID
			LOW–HIGH	−6.24	1.39	54	−4.504	**<0.001**	LOW < HIGH
			MID–HIGH	9.01	1.39	54	6.500	**<0.001**	MID > HIGH
Winter	Body size	Elevation	LOW–MID	−3.98	1.37	54	−2.908	**0.014**	LOW < MID
			LOW–HIGH	−8.98	1.37	54	−6.556	**<0.001**	LOW < HIGH
			MID–HIGH	−4.99	1.37	54	−3.648	**0.002**	MID < HIGH
Summer	Hdisp	Elevation	LOW–MID	−0.108	0.011	54	−9.547	**<0.001**	LOW < MID
			LOW–HIGH	−0.233	0.011	54	−20.637	**<0.001**	LOW < HIGH
			MID–HIGH	−0.125	0.011	54	−11.090	**<0.001**	MID < HIGH
Summer	Hdisp	Treatment	Control–OTC	0.045	0.009	54	4.913	**<0.001**	C > OTC
Summer	Hdisp	Elev:Treatment	LOW C.–MID C.	−0.086	0.016	54	−5.389	**<0.001**	LOW C. < MID C.
			LOW C.–HIGH C.	−0.246	0.016	54	−15.429	**<0.001**	LOW C. < HIGH C.
			LOW C.–LOW OTC	0.051	0.016	54	3.187	**0.028**	LOW C. < LOW OTC
			LOW C.–MID OTC	−0.079	0.016	54	−4.926	**<0.001**	LOW C. < MID OTC
			LOW C.–HIGH OTC	−0.169	0.016	54	−10.569	**<0.001**	LOW C. < HIGH OTC
			MID C.–HIGH C.	−0.160	0.016	54	−10.040	**<0.001**	MID C. < HIGH C.
			MID C.–LOW OTC	0.137	0.016	54	8.576	**<0.001**	MID C. > LOW OTC
			MID C.–MID OTC	0.007	0.016	54	0.464	0.997	MID C. = MID OTC
			MID C.–HIGH OTC	−0.083	0.016	54	−5.180	**<0.001**	MID C. < HIGH OTC
			HIGH C.–LOW OTC	0.297	0.016	54	18.616	**<0.001**	HIGH C. > LOW OTC
			HIGH C.–MID OTC	0.168	0.016	54	10.504	**<0.001**	HIGH C. > MID OTC
			HIGH C.–HIGH OTC	0.078	0.016	54	4.860	**<0.001**	HIGH C. > HIGH OTC
			LOW OTC–MID OTC	−0.130	0.016	54	−8.112	**<0.001**	LOW OTC < MID OTC
			LOW OTC–HIGH OTC	−0.220	0.016	54	−13.756	**<0.001**	LOW OTC < HIGH OTC
			MID OTC–HIGH OTC	−0.090	0.016	54	−5.644	**<0.001**	MID OTC < HIGH OTC
Winter	Hdisp	Elevation	LOW–MID	−0.101	0.019	54	−5.175	**<0.001**	LOW < MID
			LOW–HIGH	−0.188	0.019	54	−9.678	**<0.001**	LOW < HIGH
			MID–HIGH	−0.088	0.019	54	−4.504	**<0.001**	MID < HIGH
Winter	Hdisp	Treatment	Control–OTC	0.057	0.016	54	3.615	**0.001**	C > OTC
Summer	Ldisp	Elevation	LOW–MID	0.034	0.009	54	3.609	**0.002**	LOW > MID
			LOW–HIGH	0.039	0.009	54	4.223	**<0.001**	LOW > HIGH
			MID–HIGH	0.006	0.009	54	0.614	0.813	MID = HIGH
Summer	Ldisp	Treatment	Control–OTC	−0.033	0.008	54	−4.337	**0.000**	C < OTC
Summer	Ldisp	Elev:Treatment	LOW C.–MID C.	0.040	0.013	54	3.010	**0.044**	LOW C. > MID C.
			LOW C.–HIGH C.	0.066	0.013	54	4.990	**<0.001**	LOW C. > HIGH C.
			LOW C.–LOW OTC	−0.011	0.013	54	−0.863	0.954	LOW C. = LOW OTC
			LOW C.–MID OTC	0.016	0.013	54	1.231	0.820	LOW C. = MID OTC
			LOW C.–HIGH OTC	0.002	0.013	54	0.119	1.000	LOW C. = HIGH OTC
			MID C.–HIGH C.	0.026	0.013	54	1.980	0.367	MID C. = HIGH C.
			MID C.–LOW OTC	−0.051	0.013	54	−3.873	**0.004**	MID C. < LOW OTC
			MID C.–MID OTC	−0.023	0.013	54	−1.778	0.488	MID C. = MID OTC
			MID C.–HIGH OTC	−0.038	0.013	54	−2.891	0.059	MID C. = HIGH OTC
			HIGH C.–LOW OTC	−0.077	0.013	54	−5.853	**<0.001**	HIGH C. < LOW OTC
			HIGH C.–MID OTC	−0.049	0.013	54	−3.758	**0.005**	HIGH C. < MID OTC
			HIGH C.–HIGH OTC	−0.064	0.013	54	−4.870	**<0.001**	HIGH C. < HIGH OTC
			LOW OTC–MID OTC	0.028	0.013	54	2.094	0.306	LOW OTC = MID OTC
			LOW OTC–HIGH OTC	0.013	0.013	54	0.982	0.922	LOW OTC = HIGH OTC
			MID OTC–HIGH OTC	−0.015	0.013	54	−1.112	0.874	MID OTC = HIGH OTC
Winter	Ldisp	Treatment	Control–OTC	−0.039	0.013	54	−3.038	**0.004**	C < OTC
Summer	ActivDay	Elevation	LOW–MID	0.124	0.012	54	9.954	**<0.001**	LOW > MID
			LOW–HIGH	0.221	0.012	54	17.829	**<0.001**	LOW > HIGH
			MID–HIGH	0.098	0.012	54	7.875	**<0.001**	MID > HIGH
Winter	ActivDay	Elevation	LOW–MID	0.072	0.018	54	4.089	**<0.001**	LOW > MID
			LOW–HIGH	0.211	0.018	54	11.986	**<0.001**	LOW > HIGH
			MID–HIGH	0.139	0.018	54	7.897	**<0.001**	MID > HIGH
Winter	ActivDay	Treatment	Control–OTC	0.034	0.014	54	2.395	**0.020**	C > OTC
Summer	ActivNight	Elevation	LOW–MID	−0.034	0.008	54	−4.432	**<0.001**	LOW < MID
			LOW–HIGH	−0.031	0.008	54	−3.939	**0.001**	LOW < HIGH
			MID–HIGH	0.004	0.008	54	0.494	0.875	MID = HIGH
Winter	ActivNight	Elevation	LOW–MID	−0.134	0.011	54	−12.789	**<0.001**	LOW < MID
			LOW–HIGH	−0.182	0.011	54	−17.364	**<0.001**	LOW < HIGH
			MID–HIGH	−0.048	0.011	54	−4.575	**0.000**	MID < HIGH
Summer	ActivTwi	Elevation	LOW–MID	0.096	0.013	54	7.498	**<0.001**	LOW > MID
			LOW–HIGH	0.199	0.013	54	15.613	**<0.001**	LOW > HIGH
			MID–HIGH	0.103	0.013	54	8.115	**<0.001**	MID > HIGH
Winter	ActivTwi	Elevation	LOW–MID	−0.052	0.015	54	−3.417	**0.003**	LOW < MID
			LOW–HIGH	0.038	0.015	54	2.532	**0.037**	LOW > HIGH
			MID–HIGH	0.090	0.015	54	5.949	**<0.001**	MID > HIGH
Winter	ActivTwi	Treatment	Control–OTC	0.043	0.012	54	3.490	**0.001**	C > OTC

**Table A3:** Results of the ANOVA performed to investigate the effects of the elevation and the treatment on beetles functional traits including the interaction terms (Elev:Treatment) on each CWM variables. Adjusted *p* values are also presented. Significant results are marked in bold. Traits presented are Herbivore; Predator; Fungivore; Detritivore; Body size; High dispersal ability (Hdisp); Diurnal beetles (ActDay); Nocturnal beetles (ActNig); Beetles active during twilight (ActTwi); Black coloration (Black); Blackish coloration (Blackish); Reddish brown coloration (Reddish brown); and the presence of iridescence (Iridescence).

Season	Trophic Guild	Factor	Df	Sum Sq.	Mean Sq.	F Value	Pr(>F)	*p* Adjust
Summer	Herbivore	Elevation	2	1.102	0.551	99.625	**<0.001**	**<0.001**
		Treatment	1	0.003	0.003	0.585	0.448	0.562
		Elev:Treatment	2	0.033	0.016	2.968	0.060	0.104
		Residuals	54	0.299	0.006			
Winter	Herbivore	Elevation	2	0.099	0.049	12.186	**<0.001**	**<0.001**
		Treatment	1	0.003	0.003	0.826	0.367	0.481
		Elev:Treatment	2	0.011	0.006	1.395	0.257	0.385
		Residuals	54	0.219	0.004			
Summer	Predator	Elevation	2	0.071	0.036	10.111	**0.000**	**0.001**
		Treatment	1	0.000	0.000	0.012	0.915	0.969
		Elev:Treatment	2	0.005	0.002	0.666	0.518	0.622
		Residuals	54	0.190	0.004			
Winter	Predator	Elevation	2	0.052	0.026	4.172	**0.021**	**0.041**
		Treatment	1	0.085	0.085	13.641	**0.001**	**0.002**
		Elev:Treatment	2	0.008	0.004	0.627	0.538	0.652
		Residuals	54	0.336	0.006			
Summer	Fungivore	Elevation	2	0.030	0.015	23.373	**<0.001**	**<0.001**
		Treatment	1	0.004	0.004	6.674	**0.013**	**0.032**
		Elev:Treatment	2	0.001	0.001	1.09	0.344	0.468
		Residuals	54	0.034	0.001			
Winter	Fungivore	Elevation	2	0.034	0.017	8.521	**0.001**	**0.002**
		Treatment	1	0.000	0.000	0.116	0.734	0.749
		Elev:Treatment	2	0.001	0.000	0.166	0.848	0.848
		Residuals	54	0.108	0.002			
Summer	Detritivore	Elevation	2	0.001	0.001	3.459	**0.039**	0.074
		Treatment	1	0.000	0.000	0.28	0.599	0.703
		Elev:Treatment	2	0.001	0.000	1.72	0.189	0.283
		Residuals	54	0.010	0.000			
Winter	Detritivore	Elevation	2	0.011	0.006	2.104	0.132	0.217
		Treatment	1	0.077	0.077	29.021	**<0.001**	**<0.001**
		Elev:Treatment	2	0.006	0.003	1.092	0.343	0.460
		Residuals	54	0.143	0.003			
Summer	Body size	Elevation	2	203.390	101.700	116.899	**<0.001**	**<0.001**
		Treatment	1	8.170	8.170	9.390	**0.003**	**0.010**
		Elev:Treatment	2	0.390	0.200	0.225	0.799	0.891
		Residuals	54	46.980	0.870			
Winter	Body size	Elevation	2	133.800	66.900	81.485	**<0.001**	**<0.001**
		Treatment	1	12.700	12.700	15.472	**0.000**	**0.001**
		Elev:Treatment	2	0.990	0.490	0.602	0.551	0.652
		Residuals	54	44.330	0.820			
Summer	Hdisp	Elevation	2	0.032	0.016	10.984	**<0.001**	**<0.001**
		Treatment	1	0.006	0.006	4.210	**0.045**	0.084
		Elev:Treatment	2	0.003	0.002	1.081	0.347	0.468
		Residuals	54	0.079	0.001			
Winter	Hdisp	Elevation	2	0.408	0.204	51.525	**<0.001**	**<0.001**
		Treatment	1	0.006	0.006	1.419	0.239	0.369
		Elev:Treatment	2	0.009	0.005	1.140	0.327	0.460
		Residuals	54	0.214	0.004			
Summer	ActivDay	Elevation	2	0.140	0.070	35.842	**<0.001**	**<0.001**
		Treatment	1	0.010	0.010	5.138	**0.027**	0.055
		Elev:Treatment	2	0.027	0.013	6.841	**0.002**	**0.007**
		Residuals	54	0.105	0.002			
Winter	ActivDay	Elevation	2	0.609	0.305	59.960	**<0.001**	**<0.001**
		Treatment	1	0.042	0.042	8.270	**0.006**	**0.013**
		Elev:Treatment	2	0.037	0.018	3.620	**0.033**	0.061
		Residuals	54	0.274	0.005			
Summer	ActivNight	Elevation	2	0.152	0.076	47.793	**<0.001**	**<0.001**
		Treatment	1	0.005	0.005	3.274	0.076	0.124
		Elev:Treatment	2	0.009	0.005	2.977	0.059	0.104
		Residuals	54	0.086	0.002			
Winter	ActivNight	Elevation	2	1.066	0.533	204.329	**<0.001**	**<0.001**
		Treatment	1	0.015	0.015	5.724	**0.020**	**0.041**
		Elev:Treatment	2	0.027	0.013	5.150	**0.009**	**0.020**
		Residuals	54	0.141	0.003			
Summer	ActivTwi	Elevation	2	0.018	0.009	5.694	**0.006**	**0.015**
		Treatment	1	0.000	0.000	0.163	0.688	0.790
		Elev:Treatment	2	0.002	0.001	0.685	0.509	0.622
		Residuals	54	0.083	0.002			
Winter	ActivTwi	Elevation	2	0.040	0.020	6.274	**0.004**	**0.009**
		Treatment	1	0.097	0.097	30.280	**<0.001**	**<0.001**
		Elev:Treatment	2	0.010	0.005	1.590	0.213	0.340
		Residuals	54	0.174	0.003			
Summer	Black	Elevation	2	2.085	1.042	129.16	**<0.001**	**<0.001**
		Treatment	1	0.138	0.138	17.06	**<0.001**	**<0.001**
		Elev:Treatment	2	0.037	0.018	2.26	0.114	0.176
		Residuals	54	0.436	0.008			
Winter	Black	Elevation	2	0.783	0.391	71.91	**<0.001**	**<0.001**
		Treatment	1	0.125	0.125	22.948	**<0.001**	**<0.001**
		Elev:Treatment	2	0.032	0.016	2.934	0.062	0.109
		Residuals	54	0.294	0.005			
Summer	Blackish	Elevation	2	0.333	0.166	51.586	**<0.001**	**<0.001**
		Treatment	1	0.019	0.019	5.866	**0.019**	**0.041**
		Elev:Treatment	2	0.028	0.014	4.268	**0.019**	**0.041**
		Residuals	54	0.174	0.003			
Winter	Blackish	Elevation	2	0.751	0.376	54.52	**<0.001**	**<0.001**
		Treatment	1	0.002	0.002	0.268	0.607	0.688
		Elev:Treatment	2	0.196	0.098	14.257	**<0.001**	**<0.001**
		Residuals	54	0.372	0.007			
Summer	Reddish brown	Elevation	2	0.586	0.293	61.002	**<0.001**	**<0.001**
		Treatment	1	0.055	0.055	11.486	**0.001**	**0.004**
		Elev:Treatment	2	0.013	0.006	1.317	0.276	0.398
		Residuals	54	0.259	0.005			
Winter	Reddish brown	Elevation	2	0.003	0.001	0.353	0.704	0.733
		Treatment	1	0.183	0.183	43.31	**<0.001**	**<0.001**
		Elev:Treatment	2	0.065	0.032	7.664	**0.001**	**0.003**
		Residuals	54	0.228	0.004			
Summer	Iridescence	Elevation	2	0.073	0.036	24.331	**<0.001**	**<0.001**
		Treatment	1	0.010	0.010	6.604	**0.013**	**0.032**
		Elev:Treatment	2	0.009	0.004	2.882	0.065	0.109
		Residuals	54	0.080	0.001			
Winter	Iridescence	Elevation	2	0.456	0.228	89.82	**<0.001**	**<0.001**
		Treatment	1	0.022	0.022	8.679	**0.005**	**0.012**
		Elev:Treatment	2	0.019	0.009	3.71	**0.031**	0.058
		Residuals	54	0.137	0.003			

**Table A4:** Results of the post hoc pairwise comparisons performed on the different beetles’ traits when the ANOVA models were significant. Significant results are marked in bold. Traits presented are Herbivore; Predator; Fungivore; Detritivore; Body size; High dispersal ability (Hdisp); Diurnal beetles (ActDay); Nocturnal beetles (ActNig); Beetles active during twilight (ActTwi); Black coloration (Black); Blackish coloration (Blackish), Reddish-brown coloration (Reddish brown); and the presence of iridescence (Iridescence). Factors presented are the elevation, the treatment, and their interaction (Elev:Treatment). Low-elevation field (LOW); Middle-elevation field (MID); High-elevation field (HIGH); Control plots at low elevation (LOW C.); OTCs at low elevation (LOW OTC); Control plots at middle elevation (MID C.); OTCs at middle elevation (MID OTC); Control plots at high elevation (HIGH C.); OTCs at high elevation (HIGH OTC). See corresponding box plot in the main text in Figure 4.

Season	Trait	Factor	Pairwise Comparisons	Estimate	SE	df	t.Ratio	*p* Value	Trend
Summer	Herbivore	Elevation	LOW–MID	−0.275	0.024	54	−11.692	**<0.0001**	LOW < MID
			LOW–HIGH	0.024	0.024	54	1.004	0.578	LOW = HIGH
			MID–HIGH	0.299	0.024	54	12.695	**<0.0001**	MID > HIGH
Winter	Herbivore	Elevation	LOW–MID	0.099	0.020	54	4.896	**<0.0001**	LOW > MID
			LOW–HIGH	0.038	0.020	54	1.903	0.148	LOW = HIGH
			MID–HIGH	−0.060	0.020	54	−2.994	**0.011**	MID < HIGH
Summer	Predator	Elevation	LOW–MID	−0.084	0.019	54	−4.466	**<0.0001**	LOW < MID
			LOW–HIGH	−0.050	0.019	54	−2.686	0.026	LOW = HIGH
			MID–HIGH	0.033	0.019	54	1.780	0.186	MID = HIGH
Winter	Predator	Elevation	LOW–MID	−0.066	0.025	54	−2.641	**0.029**	LOW < MID
			LOW–HIGH	−0.058	0.025	54	−2.334	0.060	LOW = HIGH
			MID–HIGH	0.008	0.025	54	0.307	0.949	MID = HIGH
Winter	Predator	Treatment	Control–OTC	−0.075	0.020	54	−3.693	**0.001**	C < OTC
Summer	Fungivore	Elevation	LOW–MID	0.053	0.008	54	6.585	**<0.0001**	LOW > MID
			LOW–HIGH	0.014	0.008	54	1.700	0.215	LOW = HIGH
			MID–HIGH	−0.039	0.008	54	−4.885	**<0.0001**	MID < HIGH
Summer	Fungivore	Treatment	Control–OTC	−0.017	0.007	54	−2.583	**0.013**	C < OTC
Winter	Fungivore	Elevation	LOW–MID	0.034	0.014	54	2.395	0.052	LOW = MID
			LOW–HIGH	0.058	0.014	54	4.109	**<0.0001**	LOW > HIGH
			MID–HIGH	0.024	0.014	54	1.714	0.209	MID = HIGH
Summer	Detritivore	Elevation	LOW–MID	0.006	0.004	54	1.493	0.302	LOW = MID
			LOW–HIGH	−0.005	0.004	54	−1.129	0.501	LOW = HIGH
			MID–HIGH	−0.011	0.004	54	−2.622	**0.030**	MID < HIGH
Winter	Detritivore	Treatment	Control–OTC	0.072	0.013	54	5.387	**<0.0001**	C > OTC
Summer	Body size	Elevation	LOW–MID	−4.218	0.295	54	−14.300	**<0.001**	LOW < MID
			LOW–HIGH	−0.726	0.295	54	−2.461	**0.044**	LOW < HIGH
			MID–HIGH	3.492	0.295	54	11.838	**<0.001**	MID > HIGH
Summer	Body size	Treatment	Control–OTC	−0.738	0.241	54	−3.064	**0.003**	C < OTC
Winter	Body size	Elevation	LOW–MID	−3.233	0.287	54	−11.285	**<0.001**	LOW < MID
			LOW–HIGH	−0.136	0.287	54	−0.473	0.884	LOW = HIGH
			MID–HIGH	3.098	0.287	54	10.811	**<0.001**	MID > HIGH
Winter	Body size	Treatment	Control–OTC	−0.920	0.234	54	−3.933	**<0.001**	C < OTC
Summer	Hdsip	Elevation	LOW–MID	−0.034	0.012	54	−2.782	**0.020**	LOW < MID
			LOW–HIGH	0.023	0.012	54	1.876	0.155	LOW = HIGH
			MID–HIGH	0.056	0.012	54	4.658	**<0.001**	MID > HIGH
Summer	Hdsip	Treatment	Control–OTC	0.020	0.010	54	2.052	**0.045**	C > OTC
Winter	Hdsip	Elevation	LOW–MID	−0.138	0.020	54	−6.954	**<0.001**	LOW < MID
			LOW–HIGH	−0.197	0.020	54	−9.882	**<0.001**	LOW < HIGH
			MID–HIGH	−0.058	0.020	54	−2.928	**0.014**	MID < HIGH
Summer	ActivDay	Elevation	LOW–MID	0.101	0.014	54	7.213	**<0.001**	LOW > MID
			LOW–HIGH	0.104	0.014	54	7.446	**<0.001**	LOW > HIGH
			MID–HIGH	0.003	0.014	54	0.232	0.971	MID = HIGH
Summer	ActivDay	Treatment	Control–OTC	−0.026	0.011	54	−2.267	**0.027**	C < OTC
Summer	ActivDay	Elev:Treatment	LOW C.–MID C.	0.050	0.020	54	2.511	0.139	LOW C. = MID C.
			LOW C.–HIGH C.	0.085	0.020	54	4.289	**0.001**	LOW C. > HIGH C.
			LOW C.–LOW OTC	−0.073	0.020	54	−3.686	**0.007**	LOW C. < LOW OTC
			LOW C.–MID OTC	0.079	0.020	54	4.005	**0.003**	LOW C. > MID OTC
			LOW C.–HIGH OTC	0.050	0.020	54	2.555	0.127	LOW C. = HIGH OTC
			MID C.–HIGH C.	0.035	0.020	54	1.778	0.488	MID C. = HIGH C.
			MID C.–LOW OTC	−0.122	0.020	54	−6.197	**<0.001**	MID C. < LOW OTC
			MID C.–MID OTC	0.030	0.020	54	1.493	0.670	MID C. = MID OTC
			MID C.–HIGH OTC	0.001	0.020	54	0.044	1.000	MID C. = HIGH OTC
			HIGH C.–LOW OTC	−0.158	0.020	54	−7.975	**<0.001**	HIGH C. < LOW OTC
			HIGH C.–MID OTC	−0.006	0.020	54	−0.284	1.000	HIGH C. = MID OTC
			HIGH C.–HIGH OTC	−0.034	0.020	54	−1.734	0.516	HIGH C. = HIGH OTC
			LOW OTC–MID OTC	0.152	0.020	54	7.690	**<0.001**	LOW OTC > MID OTC
			LOW OTC–HIGH OTC	0.123	0.020	54	6.241	**<0.001**	LOW OTC > HIGH OTC
			MID OTC–HIGH OTC	−0.029	0.020	54	−1.449	0.697	MID OTC = HIGH OTC
Winter	ActivDay	Elevation	LOW–MID	0.142	0.023	54	6.309	**<0.001**	LOW > MID
			LOW–HIGH	0.246	0.023	54	10.906	**<0.001**	LOW > HIGH
			MID–HIGH	0.104	0.023	54	4.597	**<0.001**	MID > HIGH
Winter	ActivDay	Treatment	Control–OTC	0.053	0.018	54	2.876	**0.006**	C > OTC
Winter	ActivDay	Elev:Treatment	LOW C.–MID C.	0.111	0.032	54	3.470	**0.013**	LOW C. > MID C.
			LOW C.–HIGH C.	0.185	0.032	54	5.810	**<0.001**	LOW C. > HIGH C.
			LOW C.–LOW OTC	−0.009	0.032	54	−0.268	1.000	LOW C. = LOW OTC
			LOW C.–MID OTC	0.165	0.032	54	5.184	**<0.001**	LOW C. > MID OTC
			LOW C.–HIGH OTC	0.298	0.032	54	9.345	**<0.001**	LOW C. > HIGH OTC
			MID C.–HIGH C.	0.075	0.032	54	2.340	0.197	MID C. = HIGH C.
			MID C.–LOW OTC	−0.119	0.032	54	−3.738	**0.006**	MID C. < LOW OTC
			MID C.–MID OTC	0.055	0.032	54	1.714	0.529	MID C. = MID OTC
			MID C.–HIGH OTC	0.187	0.032	54	5.875	**<0.001**	MID C. > HIGH OTC
			HIGH C.–LOW OTC	−0.194	0.032	54	−6.078	**<0.001**	HIGH C. < LOW OTC
			HIGH C.–MID OTC	−0.020	0.032	54	−0.626	0.989	HIGH C. = MID OTC
			HIGH C.–HIGH OTC	0.113	0.032	54	3.536	**0.010**	HIGH C. > HIGH OTC
			LOW OTC–MID OTC	0.174	0.032	54	5.452	**<0.001**	LOW OTC > MID OTC
			LOW OTC–HIGH OTC	0.306	0.032	54	9.614	**<0.001**	LOW OTC > HIGH OTC
			MID OTC–HIGH OTC	0.133	0.032	54	4.161	**0.002**	MID OTC > HIGH OTC
Summer	ActivNight	Elevation	LOW–MID	−0.105	0.013	54	−8.334	**<0.001**	LOW < MID
			LOW–HIGH	−0.108	0.013	54	−8.594	**<0.001**	LOW < HIGH
			MID–HIGH	−0.003	0.013	54	−0.260	0.964	MID = HIGH
Winter	ActivNight	Elevation	LOW–MID	−0.209	0.016	54	−12.933	**<0.001**	LOW < MID
			LOW–HIGH	−0.322	0.016	54	−19.922	**<0.001**	LOW < HIGH
			MID–HIGH	−0.113	0.016	54	−6.988	**<0.001**	MID < HIGH
Winter	ActivNight	Treatment	Control–OTC	0.032	0.013	54	2.392	**0.020**	C > OTC
Winter	ActivNight	Elev:Treatment	LOW C.–MID C. -	0.168	0.023	54	−7.351	**<0.001**	LOW C. < MID C. -
			LOW C.–HIGH C.	−0.274	0.023	54	−11.987	**<0.001**	LOW C. < HIGH C.
			LOW C.–LOW OTC	0.091	0.023	54	3.978	**0.003**	LOW C. > LOW OTC
			LOW C.–MID OTC	−0.159	0.023	54	−6.962	**<0.001**	LOW C. < MID OTC
			LOW C.–HIGH OTC	−0.279	0.023	54	−12.209	**<0.001**	LOW C. < HIGH OTC
			MID C.–HIGH C.	−0.106	0.023	54	−4.636	**<0.001**	MID C. < HIGH C.
			MID C.–LOW OTC	0.259	0.023	54	11.328	**<0.001**	MID C. > LOW OTC
			MID C.–MID OTC	0.009	0.023	54	0.389	0.999	MID C. = MID OTC
			MID C.–HIGH OTC	−0.111	0.023	54	−4.859	**<0.001**	MID C. < HIGH OTC
			HIGH C.–LOW OTC	0.365	0.023	54	15.964	**<0.001**	HIGH C. > LOW OTC
			HIGH C.–MID OTC	0.115	0.023	54	5.025	**0.000**	HIGH C. > MID OTC
			HIGH C.–HIGH OTC	−0.005	0.023	54	−0.223	1.000	HIGH C. = HIGH OTC
			LOW OTC–MID OTC	−0.250	0.023	54	−10.940	**<0.001**	LOW OTC < MID OTC
			LOW OTC–HIGH OTC	−0.370	0.023	54	−16.187	**<0.001**	LOW OTC < HIGH OTC
			MID OTC–HIGH OTC	−0.120	0.023	54	−5.247	**<0.001**	MID OTC < HIGH OTC
Summer	ActivTwi	Elevation	LOW–MID	0.027	0.012	54	2.156	0.088	LOW = MID
			LOW–HIGH	0.041	0.012	54	3.326	**0.005**	LOW > HIGH
			MID–HIGH	0.015	0.012	54	1.171	0.476	MID = HIGH
Winter	ActivTwi	Elevation	LOW–MID	−0.050	0.018	54	−2.782	**0.020**	LOW < MID
			LOW–HIGH	−0.059	0.018	54	−3.290	**0.005**	LOW < HIGH
			MID–HIGH	−0.009	0.018	54	−0.507	0.868	MID = HIGH
Winter	ActivTwi	Treatment	Control–OTC	0.081	0.015	54	5.503	**<0.001**	C > OTC
Summer	Black	Elevation	LOW–MID	−0.452	0.028	54	−15.912	**<0.001**	LOW < MID
			LOW–HIGH	−0.170	0.028	54	−5.997	**<0.001**	LOW < HIGH
			MID–HIGH	0.282	0.028	54	9.915	**<0.001**	MID > HIGH
Summer	Black	Treatment	Control–OTC	−0.096	0.023	54	−4.130	**0.000**	C < OTC
Winter	Black	Elevation	LOW–MID	−0.140	0.023	54	−5.993	**<0.001**	LOW < MID
			LOW–HIGH	0.140	0.023	54	5.999	**<0.001**	LOW > HIGH
			MID–HIGH	0.280	0.023	54	11.993	**<0.001**	MID > HIGH
Winter	Black	Treatment	Control–OTC	−0.091	0.019	54	−4.790	**<0.001**	C < OTC
Summer	Blackish	Elevation	LOW–MID	0.181	0.018	54	10.088	**<0.001**	LOW > MID
			LOW–HIGH	0.072	0.018	54	4.021	**0.001**	LOW > HIGH
			MID–HIGH	−0.109	0.018	54	−6.067	**<0.001**	MID < HIGH
Summer	Blackish	Treatment	Control–OTC	0.036	0.015	54	2.422	**0.019**	C = OTC
Summer	Blackish	Elev:Treatment	LOW C.–MID C.	0.234	0.025	54	9.198	**<0.001**	LOW C. > MID C.
			LOW C.–HIGH C.	0.097	0.025	54	3.805	**0.005**	LOW C. > HIGH C.
			LOW C.–LOW OTC	0.087	0.025	54	3.415	**0.015**	LOW C. > LOW OTC
			LOW C.–MID OTC	0.216	0.025	54	8.485	**<0.001**	LOW C. > MID OTC
			LOW C.–HIGH OTC	0.135	0.025	54	5.298	**<0.001**	LOW C. > HIGH OTC
			MID C.–HIGH C.	−0.137	0.025	54	−5.393	**<0.001**	MID C. < HIGH C.
			MID C.–LOW OTC	−0.147	0.025	54	−5.783	**<0.001**	MID C. < LOW OTC
			MID C.–MID OTC	−0.018	0.025	54	−0.713	0.980	MID C. = MID OTC
			MID C.–HIGH OTC	−0.099	0.025	54	−3.900	**0.004**	MID C. < HIGH OTC
			HIGH C.–LOW OTC	−0.010	0.025	54	−0.389	0.999	HIGH C. = LOW OTC
			HIGH C.–MID OTC	0.119	0.025	54	4.680	**<0.001**	HIGH C. > MID OTC
			HIGH C.–HIGH OTC	0.038	0.025	54	1.493	0.670	HIGH C. = HIGH OTC
			LOW OTC–MID OTC	0.129	0.025	54	5.069	**<0.001**	LOW OTC > MID OTC
			LOW OTC–HIGH OTC	0.048	0.025	54	1.882	0.424	LOW OTC = HIGH OTC
			MID OTC–HIGH OTC	−0.081	0.025	54	−3.187	**0.027**	MID OTC < HIGH OTC
Winter	Blackish	Elevation	LOW–MID	0.084	0.026	54	3.181	**0.007**	LOW > MID
			LOW–HIGH	−0.184	0.026	54	−7.023	**<0.001**	LOW < HIGH
			MID–HIGH	−0.268	0.026	54	−10.204	**<0.001**	MID < HIGH
Winter	Blackish	Elev:Treatment	LOW C.–MID C.	0.091	0.037	54	2.457	0.155	LOW C. = MID C.
			LOW C.–HIGH C.	−0.059	0.037	54	−1.597	0.604	LOW C. = HIGH C.
			LOW C.–LOW OTC	0.077	0.037	54	2.086	0.310	LOW C. = LOW OTC
			LOW C.–MID OTC	0.153	0.037	54	4.128	**0.002**	LOW C. > MID OTC
			LOW C.–HIGH OTC	−0.232	0.037	54	−6.249	**<0.001**	LOW C. < HIGH OTC
			MID C.–HIGH C.	−0.151	0.037	54	−4.054	**0.002**	MID C. < HIGH C.
			MID C.–LOW OTC	−0.014	0.037	54	−0.372	0.999	MID C. = LOW OTC
			MID C.–MID OTC	0.062	0.037	54	1.670	0.557	MID C. = MID OTC
			MID C.–HIGH OTC	−0.323	0.037	54	−8.706	**<0.001**	MID C. < HIGH OTC
			HIGH C.–LOW OTC	0.137	0.037	54	3.683	**0.007**	HIGH C. > LOW OTC
			HIGH C.–MID OTC	0.213	0.037	54	5.724	**<0.001**	HIGH C. > MID OTC
			HIGH C.–HIGH OTC	−0.173	0.037	54	−4.652	**0.000**	HIGH C. < HIGH OTC
			LOW OTC–MID OTC	0.076	0.037	54	2.042	0.333	LOW OTC = MID OTC
			LOW OTC–HIGH OTC	−0.309	0.037	54	−8.335	**<0.001**	LOW OTC < HIGH OTC
			MID OTC–HIGH OTC	−0.385	0.037	54	−10.376	**<0.001**	MID OTC < HIGH OTC
Summer	Reddish brown	Field	LOW–MID	0.241	0.022	54	11.003	**<0.001**	LOW > MID
			LOW–HIGH	0.102	0.022	54	4.665	**0.000**	LOW > HIGH
			MID–HIGH	−0.139	0.022	54	−6.338	**<0.001**	MID < HIGH
Summer	Reddish brown	Treatment	Control–OTC	0.061	0.018	54	3.389	**0.001**	C > OTC
Winter	Reddish brown	Treatment	Control–OTC	0.110	0.017	54	6.581	**<0.001**	C > OTC
Winter	Reddish brown	Elev:Treatment	LOW C.–MID C.	0.014	0.029	54	0.469	0.997	LOW C. = Control
			LOW C.–HIGH C.	−0.076	0.029	54	−2.618	0.111	LOW C. = HIGH C.
			LOW C.–LOW OTC	0.082	0.029	54	2.809	0.071	LOW C. = LOW OTC
			LOW C.–MID OTC	0.062	0.029	54	2.131	0.287	LOW C. = MID OTC
			LOW C.–HIGH OTC	0.125	0.029	54	4.309	**0.001**	LOW C. < HIGH OTC
			MID C.–HIGH C.	−0.090	0.029	54	−3.086	0.036	MID C. = HIGH C.
			MID C.–LOW OTC	0.068	0.029	54	2.341	0.196	MID C. = LOW OTC
			MID C.–MID OTC	0.048	0.029	54	1.663	0.562	MID C. = MID OTC
			MID C.–HIGH OTC	0.112	0.029	54	3.841	**0.004**	MID C. > HIGH OTC
			HIGH C.–LOW OTC	0.158	0.029	54	5.427	**<0.001**	HIGH C. > LOW OTC
			HIGH C.–MID OTC	0.138	0.029	54	4.749	**0.000**	HIGH C. > MID OTC
			HIGH C.–HIGH OTC	0.201	0.029	54	6.927	**<0.001**	HIGH C. > HIGH OTC
			LOW OTC–MID OTC	−0.020	0.029	54	−0.678	0.984	LOW OTC = MID OTC
			LOW OTC–HIGH OTC	0.044	0.029	54	1.500	0.666	LOW OTC = HIGH OTC
			MID OTC–HIGH OTC	0.063	0.029	54	2.178	0.265	MID OTC = HIGH OTC
Summer	Iridescence	Field	LOW–MID	0.010	0.012	54	0.817	0.694	LOW = MID
			LOW–HIGH	0.078	0.012	54	6.408	**<0.001**	LOW > HIGH
			MID–HIGH	0.068	0.012	54	5.591	**<0.001**	MID > HIGH
Summer	Iridescence	Treatment	Control–OTC	−0.026	0.010	54	−2.570	**0.013**	OTC > C
Winter	Iridescence	Field	LOW–MID	0.114	0.016	54	7.178	**<0.001**	LOW > MID
			LOW–HIGH	0.213	0.016	54	13.391	**<0.001**	LOW > HIGH
			MID–HIGH	0.099	0.016	54	6.214	**<0.001**	MID > HIGH
Winter	Iridescence	Treatment	Control–OTC	−0.038	0.013	54	−2.946	**0.005**	C < OTC
Winter	Iridescence	Elev:Treatment	LOW C.–MID C.	0.085	0.023	54	3.767	**0.005**	LOW C. > MID C.
			LOW C.–HIGH C.	0.171	0.023	54	7.591	**<0.001**	LOW C. > HIGH C.
			LOW C.–LOW OTC	−0.086	0.023	54	−3.826	**0.004**	LOW C. < LOW OTC
			LOW C.–MID OTC	0.058	0.023	54	2.559	0.126	LOW C. = MID OTC
			LOW C.–HIGH OTC	0.170	0.023	54	7.522	**<0.001**	LOW C. > HIGH OTC
			MID C.–HIGH C.	0.086	0.023	54	3.824	**0.004**	MID C. > HIGH C.
			MID C.–LOW OTC	−0.171	0.023	54	−7.592	**<0.001**	MID C. < LOW OTC
			MID C.–MID OTC	−0.027	0.023	54	−1.208	0.831	MID C. = MID OTC
			MID C.–HIGH OTC	0.085	0.023	54	3.755	**0.005**	MID C. > HIGH OTC
			HIGH C.–LOW OTC	−0.257	0.023	54	−11.416	**<0.001**	HIGH C. < LOW OTC
			HIGH C.–MID OTC	−0.113	0.023	54	−5.032	**<0.001**	HIGH C. < MID OTC
			HIGH C.–HIGH OTC	−0.002	0.023	54	−0.069	1.000	HIGH C. = HIGH OTC
			LOW OTC–MID OTC	0.144	0.023	54	6.384	**<0.001**	LOW OTC > MID OTC
			LOW OTC–HIGH OTC	0.256	0.023	54	11.347	**<0.001**	LOW OTC > HIGH OTC
			MID OTC–HIGH OTC	0.112	0.023	54	4.963	**<0.001**	MID OTC > HIGH OTC
