# Supplementary material for: Unveiling Arthropod Responses to Climate Change: A Functional Trait Analysis in Intensive Pastures
Source: Insects. 2024 Sep 7;15(9):677. doi: 10.3390/insects15090677 (PMC11432249; doi:10.3390/insects15090677)
Supplement: Supplementary file 1 [file insects-15-00677-s001.zip › Supplementary Tables.pdf]

Supplementary materials

Table S1. Species collected over the winter and the summer in all sites studied. Species taxonomy and trait attributes are given.: Standardized body size (Stand. body size) in (mm); Type of food ingested Plants (FoodPl); Animals (FoodAni); Fungi (FoodFg); Detritus (FoodDet); Type of food with Plants (FoodPl); Animals (FoodAni); Fungi (FoodFg); Detritus (FoodDet); Coprophagous (FoodCopro); Mode of ingestion (IngestFood) with Chewing and cutting (IngCC); Piercing and sucking (IngPS); External digestion and sucking (IngEDS); Period of activities with Day (ActDay); Night (ActNig); Twilight (ActTwi) and Dispersal abilities (Dispersal) with High dispersal ability (Hdsip) and Low dispersal ability (Ldsip). When identification of the species or genus was not possible, the Mophospecies (MF) in given in the column "Species" (See Materials and Methods, Arthropod sampling and identification). For species lacking data, "NA" is mentioned. See Table 1 in main text for ecological relevance of the traits. See Table 1 in main text for ecological relevance of the traits.

Class	Order	Family	*Species*	Stand. body size	FoodPl	FoodAni	FoodFg	FoodDet	FoodCopro	IngestFood	ActDay	ActNig	ActTwi	DispersaLdisp
Arachnida	Araneae	Linyphiidae	*Agyneta fuscipalpa*	-0.65	0	1	0	0	0	EDS	1	1	1	Hdisp
Arachnida	Araneae	Theridiidae	*Cryptachaea blattea*	-0.48	0	1	0	0	0	EDS	0	1	0	Hdisp
Arachnida	Araneae	Dysderidae	*Dysdera crocata*	3.13	0	1	0	0	0	EDS	0	1	0	Ldisp
Arachnida	Araneae	Linyphiidae	*Erigone atra*	-0.73	0	1	0	0	0	EDS	1	1	1	Hdisp
Arachnida	Araneae	Linyphiidae	*Erigone autumnalis*	-0.98	0	1	0	0	0	EDS	1	1	1	Hdisp
Arachnida	Araneae	Linyphiidae	*Erigone dentipalpis*	-0.59	0	1	0	0	0	EDS	1	1	1	Hdisp
Arachnida	Araneae	Mimetidae	*Ero furcata*	-0.14	0	1	0	0	0	EDS	0	1	0	Ldisp
Arachnida	Araneae	Linyphiidae	Gen. sp. (MF 1264)	NA	0	1	0	0	0	EDS	0	1	0	Hdisp
Arachnida	Araneae	Gnaphosidae	*Marinarozelotes lyonneti*	1.22	0	1	0	0	0	EDS	0	1	0	Hdisp
Arachnida	Araneae	Linyphiidae	*Mermessus bryantae*	-0.83	0	1	0	0	0	EDS	1	1	1	Hdisp
Arachnida	Araneae	Linyphiidae	*Mermessus fradeorum*	-0.18	0	1	0	0	0	EDS	1	1	1	Hdisp
Arachnida	Araneae	Linyphiidae	*Neriene clathrata*	0.5	0	1	0	0	0	EDS	0	1	0	Hdisp
Arachnida	Araneae	Oecobiidae	*Oecobius navus*	-0.49	0	1	0	0	0	EDS	1	1	1	Ldisp
Arachnida	Araneae	Linyphiidae	*Oedothorax fuscus*	-0.61	0	1	0	0	0	EDS	0	1	0	Hdisp
Arachnida	Araneae	Linyphiidae	*Ostearius melanopygius*	-0.53	0	1	0	0	0	EDS	1	1	1	Hdisp
Arachnida	Araneae	Tetragnathidae	*Pachygnatha degeeri*	0.09	0	1	0	0	0	EDS	1	1	1	Hdisp

Arachnida	Araneae	Lycosidae	*Pardosa acorensis*	1.33	0	1	0	0	0	EDS	1	0	0	Hdisp
Arachnida	Araneae	Linyphiidae	*Prinerigone vagans*	-0.63	0	1	0	0	0	EDS	0	1	0	Hdisp
Arachnida	Araneae	Linyphiidae	*Tenuiphantes tenuis*	-0.52	0	1	0	0	0	EDS	0	1	0	Hdisp
Arachnida	Araneae	Gnaphosidae	*Zelotes aeneus*	1.36	0	1	0	0	0	EDS	0	1	0	Hdisp
Arachnida	Araneae	Zodariidae	*Zodarion atlanticum*	-0.28	0	1	0	0	0	EDS	1	1	1	NA
Insecta	Coleoptera	Elateridae	*Aeolus melliculus moreleti*	0.52	1	0	0	0	0	CC	0	1	0	Hdisp
Insecta	Coleoptera	Carabidae	*Agonum muelleri muelleri*	NA	0	1	0	0	0	CC	1	1	1	Hdisp
Insecta	Coleoptera	Staphylinidae	*Aleochara bipustulata*	-0.26	0	1	0	0	0	CC	0	1	0	Hdisp
Insecta	Coleoptera	Staphylinidae	*Aleochara verna*	-0.21	0	1	0	0	0	CC	0	1	0	Hdisp
Insecta	Coleoptera	Staphylinidae	*Aloconota sulcifrons*	-0.36	0	1	0	0	0	CC	0	1	0	Hdisp
Insecta	Coleoptera	Carabidae	*Amara aenea*	0.41	1	1	0	0	0	CC	1	0	0	Hdisp
Insecta	Coleoptera	Staphylinidae	*Amischa analis*	-0.5	0	1	0	0	0	CC	0	1	0	Hdisp
Insecta	Coleoptera	Staphylinidae	*Amischa forcipata*	NA	0	1	0	0	0	CC	0	1	0	Hdisp
Insecta	Coleoptera	Carabidae	*Anisodactylus binotatus*	1.02	1	1	0	0	0	CC	0	1	0	Hdisp
Insecta	Coleoptera	Staphylinidae	*Anotylus nitidifrons*	-0.65	0	1	0	0	0	CC	0	1	0	Hdisp
Insecta	Coleoptera	Staphylinidae	*Anotylus nitidulus*	-0.67	0	1	0	0	0	CC	0	1	0	Hdisp
Insecta	Coleoptera	Apionidae	*Aspidapion radiolus*	-0.65	1	0	0	0	0	CC	1	0	0	NA
Insecta	Coleoptera	Staphylinidae	*Astenus lyonessius*	-0.45	0	1	0	0	0	CC	0	1	0	Hdisp
Insecta	Coleoptera	Staphylinidae	*Atheta (Mocyta)* sp. (MF 1418)	NA	0	1	0	0	0	CC	0	1	0	Hdisp
Insecta	Coleoptera	Staphylinidae	*Atheta aeneicollis*	NA	0	1	0	0	0	CC	0	1	0	Hdisp
Insecta	Coleoptera	Staphylinidae	*Atheta fungi*	-0.62	0	0	1	0	0	CC	0	1	0	Hdisp
Insecta	Coleoptera	Staphylinidae	*Atheta palustris*	-0.58	0	1	0	0	0	CC	0	1	0	Hdisp
Insecta	Coleoptera	Staphylinidae	*Atheta pasadenae*	NA	0	1	0	0	0	CC	0	1	0	Hdisp
Insecta	Coleoptera	Carabidae	*Bembidion ambiguum*	-0.41	0	1	0	0	0	CC	1	0	0	Ldisp
Insecta	Coleoptera	Tenebrionidae	*Blaps lethifera*	3.75	1	0	0	1	0	CC	0	1	0	Ldisp
Insecta	Coleoptera	Aphodiidae	*Calamosternus granarius*	-0.13	0	0	0	0	1	CC	1	1	1	NA
Insecta	Coleoptera	Carabidae	*Calosoma olivieri*	3.37	0	1	0	0	0	CC	0	1	0	Hdisp
Insecta	Coleoptera	Staphylinidae	*Carpelimus corticinus*	-0.61	0	1	0	0	0	CC	0	1	0	Hdisp
Insecta	Coleoptera	Staphylinidae	*Carpelimus zealandicus*	NA	0	1	0	0	0	CC	0	1	0	Hdisp
Insecta	Coleoptera	Nitidulidae	*Carpophilus* sp. (MF 168)	NA	1	0	0	0	0	CC	1	0	0	Hdisp
Insecta	Coleoptera	Nitidulidae	*Carpophilus* sp. (MF 1099)	NA	1	0	0	0	0	CC	1	0	0	Hdisp
Insecta	Coleoptera	Nitidulidae	*Carpophilus fumatus*	-0.5	1	0	0	0	0	CC	1	0	0	Hdisp

Insecta	Coleoptera	Latridiidae	*Cartodere nodifer*	-0.73	0	0	1	0	0	CC	1	1	1	NA
Insecta	Coleoptera	Leiodidae	*Catops* sp. (MF 1415)	NA	0	0	0	1	0	CC	0	1	0	NA
Insecta	Coleoptera	Hydrophilidae	*Cercyon* sp. (MF 342)	NA	0	0	0	1	0	CC	1	1	1	Hdisp
Insecta	Coleoptera	Hydrophilidae	*Cercyon* sp. (MF 1005)	NA	0	0	0	1	0	CC	1	1	1	Hdisp
Insecta	Coleoptera	Hydrophilidae	*Cercyon haemorrhoidalis*	-0.51	0	0	0	1	0	CC	1	1	1	Hdisp
Insecta	Coleoptera	Chrysomelidae	Gen. sp. (MF 1623)	NA	1	0	0	0	0	CC	1	0	0	NA
Insecta	Coleoptera	Curculionidae	*Coccotrypes carpophagus*	-0.64	1	0	0	0	0	CC	1	1	1	Hdisp
Insecta	Coleoptera	Staphylinidae	*Coproporus pulchellus*	-0.7	0	1	0	0	0	CC	0	1	0	Hdisp
Insecta	Coleoptera	Staphylinidae	*Cordalia* sp. (MF 1631)	NA	0	1	0	0	0	CC	0	1	0	Hdisp
Insecta	Coleoptera	Staphylinidae	*Cordalia obscura*	-0.72	0	1	0	0	0	CC	0	1	0	Hdisp
Insecta	Coleoptera	Cryptophagidae	*Cryptophagidae*	NA	0	0	0	1	0	CC	1	1	1	NA
Insecta	Coleoptera	Cryptophagidae	*Cryptophagus* sp. (MF 145)	NA	0	0	1	0	0	CC	1	1	1	Hdisp
Insecta	Coleoptera	Cryptophagidae	*Cryptophagus* sp. (MF 146)	NA	0	0	1	0	0	CC	1	1	1	Hdisp
Insecta	Coleoptera	Dryopidae	*Dryops luridus*	-0.19	1	0	0	1	0	CC	1	1	1	NA
Insecta	Coleoptera	Chrysomelidae	*Epitrix cucumeris*	-0.72	1	0	0	0	0	CC	1	0	0	Hdisp
Insecta	Coleoptera	Chrysomelidae	*Epitrix hirtipennis*	-0.76	1	0	0	0	0	CC	1	0	0	Hdisp
Insecta	Coleoptera	Nitidulidae	*Epuraeas* sp. (MF 1271)	NA	0	0	0	1	0	CC	1	0	0	Hdisp
Insecta	Coleoptera	Nitidulidae	*Epuraea biguttata*	-0.55	1	0	1	0	0	CC	1	0	0	Hdisp
Insecta	Coleoptera	Staphylinidae	*Gabrius nigritulus*	-0.44	0	1	0	0	0	CC	0	1	0	Hdisp
Insecta	Coleoptera	Staphylinidae	*Geostiba* sp. (MF 1556)	NA	0	1	0	0	0	CC	0	1	0	Hdisp
Insecta	Coleoptera	Staphylinidae	*Gyrohypnus fracticornis*	0.32	0	1	0	0	0	CC	0	1	0	Hdisp
Insecta	Coleoptera	Carabidae	*Harpalus distinguendus distinguendus*	NA	1	1	0	0	0	CC	1	0	0	Hdisp
Insecta	Coleoptera	Anthicidae	*Hirticollis quadriguttatus*	-0.23	1	1	1	0	0	CC	1	1	1	Hdisp
Insecta	Coleoptera	Carabidae	*Laemostenus complanatus*	1.47	0	1	0	0	0	CC	0	1	0	NA
Insecta	Coleoptera	Latridiidae	Gen. sp. (MF 938)	NA	0	0	1	0	0	CC	1	1	1	NA
Insecta	Coleoptera	Mycetophagidae	*Litargus balteatus*	-0.66	0	0	1	0	0	CC	1	0	0	NA
Insecta	Coleoptera	Curculionidae	*Mecinus pascuorum*	NA	1	0	0	0	0	CC	1	0	0	NA
Insecta	Coleoptera	Elateridae	*Melanotus dichrous*	1.79	1	0	0	0	0	CC	0	1	0	NA
Insecta	Coleoptera	Nitidulidae	Gen. sp. (MF 1554)	NA	0	0	0	1	0	CC	1	0	0	NA
Insecta	Coleoptera	Carabidae	*Notiophilus quadripunctatus*	-0.2	0	1	0	0	0	CC	1	0	0	NA
Insecta	Coleoptera	Staphylinidae	*Ocypus olens*	3.61	0	1	0	0	0	CC	0	1	0	Hdisp
Insecta	Coleoptera	Staphylinidae	*Oligota pumilio*	-0.86	0	1	0	0	0	CC	0	1	0	Hdisp

Insecta	Coleoptera	Staphylinidae	*Oligota pusillima*	-0.84	0	1	0	0	0	CC	0	1	0	Hdisp
Insecta	Coleoptera	Scarabaeidae	*Onthophagus taurus*	0.71	0	0	0	0	1	CC	1	1	1	Hdisp
Insecta	Coleoptera	Scarabaeidae	*Onthophagus vacca*	0.65	0	0	0	0	1	CC	1	1	1	Hdisp
Insecta	Coleoptera	Carabidae	*Ophonus ardosiacus*	1.08	1	0	0	0	0	CC	0	1	0	NA
Insecta	Coleoptera	Curculionidae	*Orthochaetes insignis*	-0.62	1	0	0	0	0	CC	1	0	0	NA
Insecta	Coleoptera	Staphylinidae	*Oxypoda* sp. (MF 1705)	NA	0	1	0	0	0	CC	0	1	0	Hdisp
Insecta	Coleoptera	Carabidae	*Paranchus albipes*	0.82	0	1	0	0	0	CC	0	1	0	Hdisp
Insecta	Coleoptera	Phalacridae	Gen. sp. (MF 173)	NA	1	0	1	0	0	CC	1	0	0	NA
Insecta	Coleoptera	Nitidulidae	*Phenolia limbata tibialis*	0.42	1	0	1	0	0	CC	1	0	0	Hdisp
Insecta	Coleoptera	Staphylinidae	*Philonthus longicornis*	0.13	0	1	0	0	0	CC	0	1	0	Hdisp
Insecta	Coleoptera	Staphylinidae	*Philonthus quisquiliarius quisquiliarius*	0.44	0	1	0	0	0	CC	0	1	0	Hdisp
Insecta	Coleoptera	Carabidae	*Pseudoophonus rufipes*	1.58	1	1	0	0	0	CC	0	1	0	Hdisp
Insecta	Coleoptera	Staphylinidae	*Pseudoplectus perplexus*	1.92	0	1	0	0	0	CC	0	1	0	Hdisp
Insecta	Coleoptera	Ptiliidae	*Ptenidium pusillum*	-0.9	0	0	1	0	0	CC	1	1	1	Hdisp
Insecta	Coleoptera	Carabidae	*Pterostichus vernalis*	0.3	0	1	0	0	0	CC	0	1	0	Hdisp
Insecta	Coleoptera	Staphylinidae	*Quedius simplicifrons*	-0.49	0	1	0	0	0	CC	0	1	0	Hdisp
Insecta	Coleoptera	Coccinellidae	*Rhyzobius lophanthae*	-0.54	0	1	0	0	0	CC	1	0	0	NA
Insecta	Coleoptera	Staphylinidae	*Rugilus orbiculatus*	-0.31	0	1	0	0	0	CC	0	1	0	Hdisp
Insecta	Coleoptera	Scarabaeidae	Gen. sp. (MF 1518)	NA	0	0	0	1	0	CC	1	0	0	NA
Insecta	Coleoptera	Scarabaeidae	Gen. sp. (MF 1553)	NA	0	0	0	1	0	CC	1	0	0	NA
Insecta	Coleoptera	Coccinellidae	*Scymnus interruptus*	-0.68	0	1	0	0	0	CC	1	0	0	Hdisp
Insecta	Coleoptera	Coccinellidae	*Scymnus nubilus*	-0.68	0	1	0	0	0	CC	1	0	0	Hdisp
Insecta	Coleoptera	Staphylinidae	*Sepedophilus lusitanicus*	0.09	0	1	1	0	0	CC	0	1	0	Hdisp
Insecta	Coleoptera	Corylophidae	*Sericoderus lateralis*	-1	0	0	1	0	0	CC	1	0	0	Hdisp
Insecta	Coleoptera	Curculionidae	*Sitona discoideus*	-0.11	1	0	0	0	0	CC	0	0	1	Hdisp
Insecta	Coleoptera	Dryophthoridae	*Sitophilus oryzae*	-0.48	1	0	0	0	0	CC	1	0	0	NA
Insecta	Coleoptera	Hydrophilidae	*Sphaeridium bipustulatum*	-0.25	0	0	0	1	0	CC	1	1	1	Hdisp
Insecta	Coleoptera	Dryophthoridae	*Sphenophorus abbreviatus*	0.76	1	0	0	0	0	CC	1	1	1	Ldisp
Insecta	Coleoptera	Staphylinidae	Gen. sp. (MF 1632)	NA	0	1	0	0	0	CC	0	1	0	Hdisp
Insecta	Coleoptera	Staphylinidae	Gen. sp. (MF 1633)	NA	0	1	0	0	0	CC	0	1	0	Hdisp
Insecta	Coleoptera	Staphylinidae	Gen. sp. (MF 1634)	NA	0	1	0	0	0	CC	0	1	0	Hdisp
Insecta	Coleoptera	Staphylinidae	Gen. sp. (MF 1636)	NA	0	1	0	0	0	CC	0	1	0	Hdisp

Insecta	Coleoptera	Nitidulidae	*Stelidota geminata*	-0.5	1	0	0	0	0	CC	1	0	0	Hdisp
Insecta	Coleoptera	Carabidae	*Stenolophus teutonus*	0.15	0	1	0	0	0	CC	0	1	0	Hdisp
Insecta	Coleoptera	Staphylinidae	*Stenomastax madeirae*	-0.56	0	1	0	0	0	CC	0	1	0	Hdisp
Insecta	Coleoptera	Phalacridae	*Stilbus testaceus*	-0.59	1	0	1	0	0	CC	1	0	0	NA
Insecta	Coleoptera	Staphylinidae	*Sunius propinquus*	-0.51	0	1	0	0	0	CC	0	1	0	Hdisp
Insecta	Coleoptera	Staphylinidae	*Tachyporus chrysomelinus*	-0.45	0	1	0	0	0	CC	0	1	0	Hdisp
Insecta	Coleoptera	Staphylinidae	*Tachyporus nitidulus*	-0.43	0	1	0	0	0	CC	0	1	0	Hdisp
Insecta	Coleoptera	Curculionidae	*Tychius picirostris*	-0.57	1	0	0	0	0	CC	1	0	0	Hdisp
Insecta	Coleoptera	Mycetophagidae	*Typhaea stercorea*	-0.51	0	0	1	0	0	CC	1	0	0	Hdisp
Insecta	Coleoptera	Staphylinidae	*Xantholinus longiventris*	0.23	0	1	0	0	0	CC	0	1	0	Hdisp
Insecta	Dermaptera	Gen.	Gen. sp. (MF 1555)	NA	0	1	0	0	0	CC	0	1	0	NA
Insecta	Dermaptera	Anisolabididae	*Euborellia annulipes*	-1	1	1	0	1	0	CC	0	1	0	Ldisp
Insecta	Dermaptera	Forficulidae	*Forficula auricularia*	1	1	1	0	0	0	CC	0	1	0	Ldisp
Insecta	Hemiptera	Cicadellidae	*Anoscopus albifrons*	0.03	1	0	0	0	0	PS	1	0	0	Hdisp
Insecta	Hemiptera	Anthocoridae	*Anthocoris nemoralis*	-0.43	0	1	0	0	0	PS	1	0	0	NA
Insecta	Hemiptera	Rhyparochromidae	*Beosus maritimus*	1.3	1	0	0	0	0	PS	1	0	0	NA
Insecta	Hemiptera	Coccidae	Gen. sp. (MF 132)	-1.67	1	0	0	0	0	PS	1	1	1	NA
Insecta	Hemiptera	Cicadellidae	*Euscelidius variegatus*	-0.17	1	0	0	0	0	PS	1	0	0	Hdisp
Insecta	Hemiptera	Cydnidae	*Geotomus punctulatus*	0.31	1	0	0	0	0	PS	1	1	1	Hdisp
Insecta	Hemiptera	Delphacidae	*Kelisia ribauti*	-0.63	1	0	0	0	0	PS	1	0	0	NA
Insecta	Hemiptera	Lygaeidae	*Kleidocerys ericae*	0.62	1	0	0	0	0	PS	1	0	0	NA
Insecta	Hemiptera	Delphacidae	*Megamelodes quadrimaculatus*	-0.43	1	0	0	0	0	PS	1	0	0	Hdisp
Insecta	Hemiptera	Nabidae	*Nabis pseudoferus ibericus*	2.32	0	1	0	0	0	PS	1	0	0	Hdisp
Insecta	Hemiptera	Aphididae	*Rhopalosiphoninus latysiphon*	-1.26	1	0	0	0	0	PS	1	0	0	NA
Insecta	Hemiptera	Saldidae	*Saldula palustris*	-0.35	0	1	0	0	0	PS	0	1	0	Hdisp
Insecta	Hemiptera	Rhyparochromidae	*Scolopostethus decoratus*	0.36	1	0	0	0	0	PS	1	0	0	Hdisp
Insecta	Hymenoptera	Apidae	*Bombus ruderatus*	-0.21	1	0	0	0	0	PS	1	0	0	NA
Insecta	Hymenoptera	Formicidae	*Hypoponera* sp. (MF F10)	NA	1	1	1	1	1	CC	1	1	1	NA
Insecta	Hymenoptera	Formicidae	*Hypoponera eduardi*	1.85	1	1	1	1	1	CC	1	1	1	NA
Insecta	Hymenoptera	Formicidae	*Lasius grandis*	-0.88	1	1	1	1	1	CC	1	1	1	NA
Insecta	Hymenoptera	Formicidae	*Linepithema humile*	NA	1	1	1	1	1	CC	1	1	1	NA
Insecta	Hymenoptera	Formicidae	*Monomorium carbonarium*	0.11	1	1	1	1	1	CC	1	1	1	NA

Insecta	Hymenoptera	Formicidae	*Tetramorium caespitum*	-0.86	1	1	1	1	1	CC	1	1	1	NA
Diplopoda	Julida	Blaniulidae	*Blaniulus guttulatus*	-0.95	1	0	0	1	0	CC	0	1	0	Ldisp
Diplopoda	Julida	Julidae	*Cylindroiulus propinquus*	1.13	0	0	0	1	0	CC	0	1	0	Ldisp
Diplopoda	Julida	Blaniulidae	*Nopoiulus kochii*	-0.83	1	0	0	1	0	CC	0	1	0	NA
Diplopoda	Julida	Julidae	*Ommatoiulus moreleti*	1.31	1	0	0	1	0	CC	0	1	0	Ldisp
Diplopoda	Julida	Blaniulidae	*Proteroiulus fuscus*	-0.65	1	0	0	1	0	CC	0	1	0	Ldisp
Insecta	Lepidoptera	Noctuidae	*Agrotis* sp. (MF 269)	NA	1	0	0	0	0	CC	0	1	0	Ldisp
Insecta	Lepidoptera	Noctuidae	*Mythimna unipuncta*	-1	1	0	0	0	0	CC	0	1	0	Hdisp
Insecta	Lepidoptera	Noctuidae	Gen. sp. (MF 345)	1	1	0	0	0	0	CC	0	1	0	NA
Chilipoda	Lithobiomorpha	Lithobiidae	*Lithobius* sp. (MF 1006)	-1	0	1	0	0	0	CC	0	1	0	Ldisp
Chilipoda	Lithobiomorpha	Lithobiidae	*Lithobius pilicornis pilicornis*	1	0	1	0	0	0	CC	0	1	0	Ldisp
Insecta	Neuroptera	Chrysopidae	*Chrysoperla lucasina*	0.71	1	1	0	0	0	CC	0	1	1	NA
Insecta	Neuroptera	Hemerobiidae	Gen. sp. (MF 954)	-1.41	0	1	0	0	0	CC	0	1	0	NA
Arachnida	Opiliones	Sclerosomatidae	*Homalenotus coriaceus*	-1	1	1	1	1	1	CC	0	1	0	Ldisp
Arachnida	Opiliones	Leiobunidae	*Leiobunum blackwalli*	1	1	1	1	1	1	CC	0	1	0	Hdisp
Insecta	Orthoptera	Gryllidae	*Eumodicogryllus bordigalensis*	-1	1	0	0	0	0	CC	0	1	0	Hdisp
Insecta	Orthoptera	Gryllidae	*Gryllus bimaculatus*	1	1	1	0	0	0	CC	1	1	0	Hdisp
Diplopoda	Polydesmida	Paradoxosomatidae	*Oxidus gracilis*	-1	0	0	0	1	0	CC	0	1	0	Ldisp
Diplopoda	Polydesmida	Polydesmidae	*Polydesmus coriaceus*	1	0	0	0	1	0	CC	0	1	0	Ldisp
Arachnida	Pseudoscorpiones	Chthoniidae	*Chthonius ischnocheles*	-1	0	1	0	0	0	CC	1	1	1	Ldisp
Arachnida	Pseudoscorpiones	Neobisiidae	*Neobisium maroccanum*	1	0	1	0	0	0	CC	1	1	1	NA
Insecta	Psocodea	Ectopsocidae	*Ectopsocus briggsi*	2	1	0	1	0	0	CC	1	1	1	Hdisp
Insecta	Psocodea	Gen.	Gen. sp. (MF 1693)	NA	1	0	0	1	0	CC	1	1	1	NA
Chilipoda	Scutigeromorpha	Scutigeridae	*Scutigera coleoptrata*	12.64	0	1	0	0	0	CC	0	1	0	Ldisp

Table S2. Beetle species collected over the winter and the summer in all sites studied. Species taxonomy and trait attributes are given. Body size (Stand. body size) in (mm); Type of food ingested Plants (FoodPl); Animals (FoodAni); Fungi (FoodFg); Detritus (FoodDet); Type of food with Plants (FoodPl); Animals (FoodAni); Fungi (FoodFg); Detritus (FoodDet); Coprophagous (FoodCopro); Period of activities with Day (ActDay); Night (ActNig); Twilight (ActTwi); Dispersal abilities (Dispersal) with High dispersal ability (Hdsip) and Low dispersal ability (Ldsip); Presence (1) or absence (0) of Iridescence and Cuticle color (Color). When identification of the species or genus was not possible, the Mophospecies (MF) in given in the column "Species" (See Materials and Methods, Arthropod sampling and identification). For species lacking data, "NA" is mentioned. See Table 1 in main text for ecological relevance of the traits.

Class	order	family	Species	bodysize	FoodPl	FoodAni	FoodFg	FoodDet	FoodCopro	ActDay	ActNig	ActTwi	Dispersal	Iridescence	Color
Insecta	Coleoptera	Hydrophilidae	*Cercyon* .sp	NA	0	0	0	1	0	1	1	1	Hdsip	0	ReddishBrown
Insecta	Coleoptera	Scarabaeidae	*Onthophagus vacca*	7.00	0	0	0	0	1	1	1	1	Hdsip	1	ReddishBrown
Insecta	Coleoptera	Tenebrionidae	*Blaps lethifera*	19.17	1	0	0	1	0	0	1	0	Ldisp	0	Black
Insecta	Coleoptera	Staphylinidae	*Amischa forcipata*	NA	0	1	0	0	0	0	1	0	Hdsip	0	Blackish
Insecta	Coleoptera	Nitidulidae	*Carpophilus* .sp	NA	1	0	0	0	0	1	0	0	Hdsip	0	Blackish
Insecta	Coleoptera	Aphodiidae	*Calamosternus granarius*	3.94	0	0	0	0	1	1	1	1	NA	0	Blackish
Insecta	Coleoptera	Carabidae	*Notiophilus quadripunctatus*	3.64	0	1	0	0	0	1	0	0	NA	1	Black
Insecta	Coleoptera	Mycetophagidae	*Typhaea stercorea*	2.43	0	0	1	0	0	1	0	0	Hdsip	0	ReddishBrown
Insecta	Coleoptera	Curculionidae	*Coccotrypes carpophagus*	1.94	1	0	0	0	0	1	1	1	Hdsip	0	ReddishBrown
Insecta	Coleoptera	Nitidulidae	*Epuraea* .sp	NA	0	0	0	1	0	1	0	0	Hdsip	0	ReddishBrown
Insecta	Coleoptera	Staphylinidae	*Carpelimus zealandicus*	NA	0	1	0	0	0	0	1	0	Hdsip	0	Blackish
Insecta	Coleoptera	Leiodidae	*Catops* .sp	NA	0	0	0	1	0	0	1	0	NA	0	ReddishBrown
Insecta	Coleoptera	Staphylinidae	*Atheta (Mocyta)*	NA	0	1	0	0	0	0	1	0	Hdsip	0	NA
Insecta	Coleoptera	Staphylinidae	*Tachyporus chrysomelinus*	2.68	0	1	0	0	0	0	1	0	Hdsip	0	ReddishBrown
Insecta	Coleoptera	Staphylinidae	*Oligota pusillima*	1.12	0	1	0	0	0	0	1	0	Hdsip	0	Blackish
Insecta	Coleoptera	Hydrophilidae	*Sphaeridium bipustulatum*	3.45	0	0	0	1	0	1	1	1	Hdsip	0	Blackish
Insecta	Coleoptera	Cryptophagidae	*Cryptophagus* .sp	NA	0	0	1	0	0	1	1	1	Hdsip	0	ReddishBrown
Insecta	Coleoptera	Cryptophagidae	*Cryptophagus* .sp	NA	0	0	1	0	0	1	1	1	Hdsip	0	ReddishBrown
Insecta	Coleoptera	Dryopidae	*Dryops luridus*	3.71	1	0	0	1	0	1	1	1	NA	0	Blackish
Insecta	Coleoptera	Scarabaeidae	*X*	NA	0	0	0	1	0	1	0	0	NA	0	NA
Insecta	Coleoptera	Scarabaeidae	*X*	NA	0	0	0	1	0	1	0	0	NA	0	NA
Insecta	Coleoptera	Nitidulidae	*X*	NA	0	0	0	1	0	1	0	0	NA	0	ReddishBrown
Insecta	Coleoptera	Staphylinidae	*Geostiba* sp.	NA	0	1	0	0	0	0	1	0	Hdsip	0	ReddishBrown

Insecta	Coleoptera	Staphylinidae	*Atheta fungi*	1.99	0	0	1	0	0	0	1	0	Hdsip	0	Blackish
Insecta	Coleoptera	Carabidae	*Amara aenea*	6.04	1	1	0	0	0	1	0	0	Hdsip	1	Black
Insecta	Coleoptera	Carabidae	*Bembidion ambiguum*	2.83	0	1	0	0	0	1	0	0	Ldisp	1	Black
Insecta	Coleoptera	Chrysomelidae	*X*	NA	1	0	0	0	0	1	0	0	NA	0	NA
Insecta	Coleoptera	Staphylinidae	*Cordalia* sp.	NA	0	1	0	0	0	0	1	0	Hdsip	0	Blackish
Insecta	Coleoptera	Staphylinidae	*X*	NA	0	1	0	0	0	0	1	0	Hdsip	0	NA
Insecta	Coleoptera	Staphylinidae	*X*	NA	0	1	0	0	0	0	1	0	Hdsip	0	NA
Insecta	Coleoptera	Staphylinidae	*X*	NA	0	1	0	0	0	0	1	0	Hdsip	0	NA
Insecta	Coleoptera	Staphylinidae	*X*	NA	0	1	0	0	0	0	1	0	Hdsip	0	NA
Insecta	Coleoptera	Curculionidae	*Tychius picirostris*	2.18	1	0	0	0	0	1	0	0	Hdsip	0	ReddishBrown
Insecta	Coleoptera	Staphylinidae	*Aleochara verna*	3.63	0	1	0	0	0	0	1	0	Hdsip	0	Blackish
Insecta	Coleoptera	Staphylinidae	*Oxypoda* sp.	NA	0	1	0	0	0	0	1	0	Hdsip	0	Blackish
Insecta	Coleoptera	Cryptophagidae	*Cryptophagidae*	NA	0	0	0	1	0	1	1	1	NA	0	ReddishBrown
Insecta	Coleoptera	Staphylinidae	*Philonthus longicornis*	4.95	0	1	0	0	0	0	1	0	Hdsip	0	ReddishBrown
Insecta	Coleoptera	Phalacridae	*X*	NA	1	0	1	0	0	1	0	0	NA	0	Blackish
Insecta	Coleoptera	Staphylinidae	*Gabrius nigritulus*	2.71	0	1	0	0	0	0	1	0	Hdsip	0	Blackish
Insecta	Coleoptera	Chrysomelidae	*Epitrix cucumeris*	1.62	1	0	0	0	0	1	0	0	Hdsip	0	Black
Insecta	Coleoptera	Nitidulidae	*Carpophilus fumatus*	2.49	1	0	0	0	0	1	0	0	Hdsip	0	ReddishBrown
Insecta	Coleoptera	Staphylinidae	*Aleochara bipustulata*	3.41	0	1	0	0	0	0	1	0	Hdsip	0	Blackish
Insecta	Coleoptera	Staphylinidae	*Atheta palustris*	2.16	0	1	0	0	0	0	1	0	Hdsip	0	ReddishBrown
Insecta	Coleoptera	Staphylinidae	*Rugilus orbiculatus*	3.22	0	1	0	0	0	0	1	0	Hdsip	0	Blackish
Insecta	Coleoptera	Staphylinidae	*Anotylus nitidifrons*	1.89	0	1	0	0	0	0	1	0	Hdsip	0	ReddishBrown
Insecta	Coleoptera	Staphylinidae	*Xantholinus longiventris*	5.33	0	1	0	0	0	0	1	0	Hdsip	1	Black
Insecta	Coleoptera	Nitidulidae	*Carpophilus* sp.	NA	1	0	0	0	0	1	0	0	Hdsip	0	Blackish
Insecta	Coleoptera	Carabidae	*Agonum muelleri muelleri*	NA	0	1	0	0	0	1	1	1	Hdsip	1	Black
Insecta	Coleoptera	Elateridae	*Aeolus melliculus moreleti*	6.5	1	0	0	0	0	0	1	0	Hdsip	0	ReddishBrown
Insecta	Coleoptera	Dryophthoridae	*Sitophilus oryzae*	2.57	1	0	0	0	0	1	0	0	NA	0	ReddishBrown
Insecta	Coleoptera	Nitidulidae	*Stelidota geminata*	2.49	1	0	0	0	0	1	0	0	Hdsip	0	ReddishBrown
Insecta	Coleoptera	Carabidae	*Pterostichus vernalis*	5.63	0	1	0	0	0	0	1	0	Hdsip	0	Black
Insecta	Coleoptera	Elateridae	*Melanotus dichrous*	11.46	1	0	0	0	0	0	1	0	NA	0	Blackish
Insecta	Coleoptera	Mycetophagidae	*Litargus balteatus*	1.85	0	0	1	0	0	1	0	0	NA	0	ReddishBrown
Insecta	Coleoptera	Hydrophilidae	*Cercyon* sp.	NA	0	0	0	1	0	1	1	1	Hdsip	0	ReddishBrown

Insecta	Coleoptera	Staphylinidae	*Quedius simplicifrons*	2.52	0	1	0	0	0	0	1	0	Hdsip	0	Blackish
Insecta	Coleoptera	Curculionidae	*Sitona discoideus*	4.00	1	0	0	0	0	0	0	1	Hdsip	0	Greyish
Insecta	Coleoptera	Curculionidae	*Orthochaetes insignis*	2.00	1	0	0	0	0	1	0	0	NA	0	ReddishBrown
Insecta	Coleoptera	Staphylinidae	*Carpelimus corticinus*	2.03	0	1	0	0	0	0	1	0	Hdsip	0	Blackish
Insecta	Coleoptera	Carabidae	*Calosoma olivieri*	17.67	0	1	0	0	0	0	1	0	Hdsip	1	Black
Insecta	Coleoptera	Hydrophilidae	*Cercyon haemorrhoidalis*	2.45	0	0	0	1	0	1	1	1	Hdsip	0	ReddishBrown
Insecta	Coleoptera	Carabidae	*Laemostenus complanatus*	10.22	0	1	0	0	0	0	1	0	NA	0	Black
Insecta	Coleoptera	Carabidae	*Anisodactylus binotatus*	8.43	1	1	0	0	0	0	1	0	Hdsip	0	Black
Insecta	Coleoptera	Nitidulidae	*Phenolia limbata tibialis*	6.1	1	0	1	0	0	1	0	0	Hdsip	0	Blackish
Insecta	Coleoptera	Staphylinidae	*Sunius propinquus*	2.44	0	1	0	0	0	0	1	0	Hdsip	0	ReddishBrown
Insecta	Coleoptera	Staphylinidae	*Pseudoplectus perplexus*	12.00	0	1	0	0	0	0	1	0	Hdsip	0	Blackish
Insecta	Coleoptera	Carabidae	*Paranchus albipes*	7.65	0	1	0	0	0	0	1	0	Hdsip	0	Blackish
Insecta	Coleoptera	Staphylinidae	*Cordalia obscura*	1.61	0	1	0	0	0	0	1	0	Hdsip	0	Blackish
Insecta	Coleoptera	Dryophthoridae	*Sphenophorus abbreviatus*	7.43	1	0	0	0	0	1	1	1	Ldisp	0	Blackish
Insecta	Coleoptera	Anthicidae	*Hirticollis quadriguttatus*	3.53	1	1	1	0	0	1	1	1	Hdsip	0	BlackYellow
Insecta	Coleoptera	Staphylinidae	*Gyrohypnus fracticornis*	5.68	0	1	0	0	0	0	1	0	Hdsip	0	Black
Insecta	Coleoptera	Staphylinidae	*Atheta aeneicollis*	NA	0	1	0	0	0	0	1	0	Hdsip	0	Blackish
Insecta	Coleoptera	Nitidulidae	*Epuraea biguttata*	2.28	1	0	1	0	0	1	0	0	Hdsip	0	ReddishBrown
Insecta	Coleoptera	Coccinellidae	*Scymnus interruptus*	1.76	0	1	0	0	0	1	0	0	Hdsip	0	Blackish
Insecta	Coleoptera	Coccinellidae	*Scymnus nubilus*	1.78	0	1	0	0	0	1	0	0	Hdsip	0	ReddishBrown
Insecta	Coleoptera	Corylophidae	*Sericoderus lateralis*	0.5	0	0	1	0	0	1	0	0	Hdsip	0	ReddishBrown
Insecta	Coleoptera	Staphylinidae	*Amischa analis*	2.46	0	1	0	0	0	0	1	0	Hdsip	0	ReddishBrown
Insecta	Coleoptera	Coccinellidae	*Rhyzobius lophanthae*	2.3	0	1	0	0	0	1	0	0	NA	1	Blackish
Insecta	Coleoptera	Curculionidae	*Mecinus pascuorum*	NA	1	0	0	0	0	1	0	0	NA	0	Blackish
Insecta	Coleoptera	Staphylinidae	*Sepedophilus lusitanicus*	4.79	0	1	1	0	0	0	1	0	Hdsip	0	Blackish
Insecta	Coleoptera	Scarabaeidae	*Onthophagus taurus*	7.23	0	0	0	0	1	1	1	1	Hdsip	0	Black
Insecta	Coleoptera	Carabidae	*Stenolophus teutonus*	5.01	0	1	0	0	0	0	1	0	Hdsip	0	ReddishBrown
Insecta	Coleoptera	Phalacridae	*Stilbus testaceus*	2.11	1	0	1	0	0	1	0	0	NA	0	ReddishBrown
Insecta	Coleoptera	Latridiidae	*Cartodere nodifer*	1.56	0	0	1	0	0	1	1	1	NA	0	ReddishBrown
Insecta	Coleoptera	Ptiliidae	*Ptenidium pusillum*	0.89	0	0	1	0	0	1	1	1	Hdsip	0	Blackish
Insecta	Coleoptera	Carabidae	*Pseudoophonus rufipes*	10.66	1	1	0	0	0	0	1	0	Hdsip	0	Black
Insecta	Coleoptera	Carabidae	*Harpalus distinguendus distinguendus*	NA	1	1	0	0	0	1	0	0	Hdsip	1	Black

Insecta	Coleoptera	Staphylinidae	*Philonthus quisquiliarius quisquiliarius*	6.15	0	1	0	0	0	0	1	0	Hdsip	1	Blackish
Insecta	Coleoptera	Staphylinidae	*Oligota pumilio*	1.04	0	1	0	0	0	0	1	0	Hdsip	0	Blackish
Insecta	Coleoptera	Staphylinidae	*Astenus lyonessius*	2.68	0	1	0	0	0	0	1	0	Hdsip	0	ReddishBrown
Insecta	Coleoptera	Carabidae	*Ophonus ardosiacus*	8.7	1	0	0	0	0	0	1	0	NA	1	Blackish
Insecta	Coleoptera	Apionidae	*Aspidapion radiolus*	1.9	1	0	0	0	0	1	0	0	NA	1	Black
Insecta	Coleoptera	Staphylinidae	*Coproporus pulchellus*	1.69	0	1	0	0	0	0	1	0	Hdsip	0	ReddishBrown
Insecta	Coleoptera	Staphylinidae	*Atheta pasadenae*	NA	0	1	0	0	0	0	1	0	Hdsip	0	ReddishBrown
Insecta	Coleoptera	Staphylinidae	*Stenomastax madeirae*	2.25	0	1	0	0	0	0	1	0	Hdsip	0	Blackish
Insecta	Coleoptera	Chrysomelidae	*Epitrix hirtipennis*	1.46	1	0	0	0	0	1	0	0	Hdsip	0	ReddishBrown
Insecta	Coleoptera	Staphylinidae	*Aloconota sulcifrons*	3.02	0	1	0	0	0	0	1	0	Hdsip	0	Blackish
Insecta	Coleoptera	Staphylinidae	*Ocypus olens*	18.63	0	1	0	0	0	0	1	0	Hdsip	0	Black
Insecta	Coleoptera	Staphylinidae	*Anotylus nitidulus*	1.79	0	1	0	0	0	0	1	0	Hdsip	0	Blackish
Insecta	Coleoptera	Staphylinidae	*Tachyporus nitidulus*	2.73	0	1	0	0	0	0	1	0	Hdsip	0	Black
Insecta	Coleoptera	Latridiidae	*X*	NA	0	0	1	0	0	1	1	1	NA	0	ReddishBrown
